# Supplementary material for: Expanding the Scope of Orthogonal Translation with Pyrrolysyl-tRNA Synthetases Dedicated to Aromatic Amino Acids
Source: Molecules. 2020 Sep 25;25(19):4418. doi: 10.3390/molecules25194418 (PMC7582959; doi:10.3390/molecules25194418)
Supplement: Supplementary file 1 [file molecules-25-04418-s001.pdf]

Expanding the Scope of Orthogonal Translation with Pyrrolysyl-tRNA Synthetases Dedicated to Aromatic Amino Acids

Supplementary Information

Hsueh-Wei Tseng [1], Tobias Baumann [1], Huan Sun [1], Yane-Shih Wang [2,3], Zoya Ignatova [4], and Nediljko Budisa [1,5,*]

[1] Institut für Chemie, Technische Universität Berlin, Müller-Breslau-Straße 10, 10623 Berlin, Germany; justin3926@hotmail.com (H.W.T.); tobias.baumann@tu-berlin.de (T.B.); huansunluck@gmail.com (H.S.); nediljko.budisa@tu-berlin.de (N.B.).

[2] Institute of Biological Chemistry, Academia Sinica, Taipei, Taiwan; yaneshihwang@gate.sinica.edu.tw (Y.S.W.)

[3] Institute of Biochemical Sciences, National Taiwan University, Taipei, Taiwan

[4] Institute of Biochemistry and Molecular Biology, University of Hamburg, 20146 Hamburg, Germany; zoya.ignatova@uni-hamburg.de (Z.I.)

[5] Department of Chemistry, University of Manitoba, Winnipeg, MB R3T 2N2, Canada; nediljko.budisa@umanitoba.ca (N.B.)

* Correspondence: nediljko.budisa@tu-berlin.de or nediljko.budisa@umanitoba.ca; Tel.: +49-30-314-28821 or +1-204-474-9178 (N.B.)

Note S1. DNA sequences

Sequence of the *Mm*PylRS gene used in this study. Mutation sites for expanding the aaRS substrate range (L305, Y306, L309, N346, C348, V401, and W417) highlighted in yellow; mutation sites for improving the aaRS efficiency (T13, V31, I36, T56, R61, H62, H63, A100, and S193) highlighted in green.

atggataaaaaaccactaaacactctgatatctgcaaccgggctctggatgtccaggaccggaacaattcataaaataaaacaccacgaagtctctcgaagcaaaa
tctatattgaaatggcatgcggagaccaccttgttgtaaacaactccaggagcagcaggactgcaagagcgctcaggcaccacaaatacaggaagacctgcaaac
gctgcagggtttcggatgaggatctcaataagttcctcacaaaggcaaacgaagaccagacaagcgtaaaagtcaaggtcgtttctgcccctaccagaacgaaaa
aggcaatgccaaaatccgttgcgagagccccgaaacctcttgagaatacagaagcggcacaggctcaaccttctggatctaaattttcacctgcgataccggtttcc
acccaagagtcagtttctgtcccggcatctgtttcaacatcaatatcaagcatttctacaggagcaactgcatccgcactggtaaaagggaatacgaaccccattaca
tccatgtctgcccctgttcaggcaagtgcccccgcacttacgaagagccagactgacaggcttgaagtcctgttaaacccaaaagatgagatttccctgaattccgg
caagcctttcagggagcttgagtccgaattgctctctcgcagaaaaaaagacctgcagcagatctacgcggaagaaagggagaattatctggggaaactcgagc
gtgaaattaccaggttctttgtggacaggggttttctggaaataaaatccccgatcctgatccctcttgagtatatcgaaaggatgggcattgataatgataccgaa
ctttcaaaacagatcttcagggttgacaagaacttctgcctgagacccatgcttgctccaaacctttacaactacctgcgcaagcttgacagggccctgcctgatcca
ataaaaatttttgaaataggcccatgctacagaaaagagtccgacggcaaagaacacctcgaagagtttaccatgctgaacttctgccagatgggatcgggatgc
acacgggaaaatcttgaaagcataattacggacttcctgaaccacctgggaattgatttcaagatcgtaggcgattcctgcatggtctatggggataccttgatgt
aatgcacggagacctggaactttcctctgcagtagtcggacccataccgcttgaccgggaatggggtattgataaaccctggataggggcaggtttcgggctcga
acgccttctaaaggttaaacacgactttaaaaatatcaagagagctgcaaggtccgagtcttactataacgggatttctaccaacctgtaa

Sequence of the codon-optimized *Mm*PylRS gene used in this study.

atggataaaaaaccactaaacactctgatctctgctactggtctgtggatgagtcgtaccggaaccattcataaaatcaaacaccacgaggttagccgttcgaaaat
ctatattgagatggcgtgtggcgatcatctggttgtgaacaatagccgctcttctcgtacagcacgtgcactgcgtcaccacaaatatcgtaaaacctgtaaacgtt
gccgtgtgtccgatgaggatctgaacaaattcctgacaaaagccaatgaggaccaaacaagcgtgaaagtgaaagtcgttagcgctcctacccgtactaaaaaag
caatgccgaaatccgttgctcgtgcccctaaaccactggaaaacactgaagcagcacaggcacagccgtctggaagcaaattctctccggccattcctgtttctacc
caggagtccgtttctgttccagcaagtgtgagcaccagcattagcagtattagcaccggtgccaccgctagcgccctggttaaaggcaataccaatccgattacaa
gcatgtctgccccggttcaagcatcagctccagcactgacaaaatcccaaaccgatcgtctggaggttctgctgaatccgaaagacgaaatcagcctgaattccgg
caaaccgtttcgtgaactggagagcgaactgctgtcacgtcgtaaaaaagacctgcaacaaatctatgccgaagaacgtgagaactatctggggaaactggaac
gtgaaatcacccgctttttcgtggatcgtggctttctggagatcaaatccccgattctgattcctctggagtatatcgagcgtatgggcatcgacaatgataccgaac
tgagcaaacaaattttccgtgtggataaaaacttctgtctgcgccctatgctggcaccaaatctgtataactatctgcgcaaactggaccgtgccctgcctgatcctat
caaaatcttcgagatcggcccgtgttatcgtaaagagtccgacggtaaagaacatctggaggagtttaccatgctgaacttttgccaaatgggttcaggttgtactc
gtgagaacctggaaagcatcatcaccgatttctgaaccacctgggcattgacttcaaaattgtgggcgacagctgtatggtgtatggcgacaccctggatgtcat
gcacggcgacctggaactgtctagtgccgttgttggaccaattccgctggaccgtgagtggggtatcgacaaaccgtggatcggagcaggattcggtctggaac
gcctgctgaaagtgaaacacgacttcaaaaacatcaaacgtgccgcccgttctgaatcgtattataacgggatttctaccaacctgtaa

Sequence of the stop codon suppression reporter sfGFP-R2TAG with the in-frame amber stop codon (sfGFP position 2) highlighted in red.

atgtagaaaggcgaagagctgttcactggtgtcgtccctattctggtggaactggatggtgatgtcaacggtcataagttttccgtgcgtggcgagggtgaaggt
gacgcaactaatggtaaactgacgctgaagttcatctgtactactggtaaactgccggtaccttggccgactctggtaacgacgctgacttatggtgttcagtgcttt
gctcgttatccggaccatatgaagcagcatgacttcttcaagtccgccatgccggaaggctatgtgcaggaacgcacgatttcctttaaggatgacggcacgtaca
aaacgcgtgcggaagtgaaatttgaaggcgataccctggtaaaccgcattgagctgaaaggcattgactttaaagaagacggcaatatcctgggccataagctg
gaatacaattttaacagccacaatgtttacatcaccgccgataaacaaaaaaatggcattaaagcgaattttaaaattcgccacaacgtggaggatggcagcgtgc
agctggctgatcactaccagcaaaacactccaatcggtgatggtcctgttctgctgccagacaatcactatctgagcacgcaaagcgttctgtctaaagatccgaac
gagaaacgcgatcatatggttctgctggagttcgtaaccgcagcgggcatcacgcatggtatggatgaactgtacaaaggcagccatcatcatcatcatcactaa

Note S2. Primer list

Name	Sequence (5´->3´)
PylRS_pBU_F	AGAGGCATATGGATAAAAAACCACTAAACACTCTG
PylRS_pBU_R	AGAGGGGTACCAAAGCAGAAAAAACGCCGCTGAAC
*Mm*PylRS-N346G/C348Q_F	CACCTCGAAGAGTTTACCATGCTGGGCTTCCAGCAGATGGGATCGG
*Mm*PylRS-N346G/C348Q_R	CCGTGTGCATCCCGATCCCATCTGCTGGAAGCCCAGCATGGTAAACTC
*Mm*PylRS-N346A/C348Q_F	CACCTCGAAGAGTTTACCATGCTGGCGTTCCAGCAGATGGGATCGG
*Mm*PylRS-N346A/C348Q_R	CCGTGTGCATCCCGATCCCATCTGCTGGAACGCCAGCATGGTAAACTC
*Mm*PylRS-N346S/C348Q_F	CACCTCGAAGAGTTTACCATGCTGAGCTTCCAGCAGATGGGATCGG
*Mm*PylRS-N346S/C348Q_R	CCGTGTGCATCCCGATCCCATCTGCTGGAAGCTCAGCATGGTAAACTC
*Mm*PylRS-N346S/C348M_F	CACCTCGAAGAGTTTACCATGCTGAGCTTCATGCAGATGGGATCGG
*Mm*PylRS-N346S/C348M_R	CCGTGTGCATCCCGATCCCATCTGCATGAAGCTCAGCATGGTAAACTC
*Mm*PylRS-N346G/C348M_F	CACCTCGAAGAGTTTACCATGCTGGGCTTCATGCAGATGGGATCGG
*Mm*PylRS-N346G/C348M_R	CCGTGTGCATCCCGATCCCATCTGCATGAAGCCCAGCATGGTAAACTC
*Mm*PylRS-N346A/C348M_F	CACCTCGAAGAGTTTACCATGCTGGCGTTCATGCAGATGGGATCGG
*Mm*PylRS-N346A/C348M_R	CCGTGTGCATCCCGATCCCATCTGCATGAACGCCAGCATGGTAAACTC
*Mm*PylRS-L305M/Y306L/L309A_F	GACCCATGCTTGCTCCAAACATGCTGAACTACGCGCGCAAGCTTG
*Mm*PylRS-L305M/Y306L/L309A_R	GGGCCCTGTCAAGCTTGCGCGCGTAGTTCAGCATGTTTGGAGCAAGC
*Mm*PylRS-L305M/Y306L/L309S_F	GACCCATGCTTGCTCCAAACATGCTGAACTACAGCCGCAAGCTTG
*Mm*PylRS-L305M/Y306L/L309S_R	GGGCCCTGTCAAGCTTGCGGCTGTAGTTCAGCATGTTTGGAGCAAGC
*Mm*PylRS-W417NNK_F	GAATGGGGTATTGATAAACCCNNKATAGGGGCAGGTTTCG
*Mm*PylRS-W417NNK_R	TTCGAGCCCGAAACCTGCCCCTATMNNGGGTTTATCAATAC
*Mm*PylRS-V401NNK_F	GACCTGGAACTTTCCTCTGCANNKGTCGGACCCATACCG
*Mm*PylRS-V401NNK_R	CCGGTCAAGCGGTATGGGTCCGACMNNTGCAGAGGAAAG
*Mm*PylRS-S193NNK_F	CAGGCAAGTGCCCCCGCACTTACGAAGNNKCAGACTGACAG
*Mm*PylRS-S193NNK_R	GACTTCAAGCCTGTCAGTCTGMNNCTTCGTAAGTGCG
*Mm*PylRS-T13I_NdeI_F	AGAGGCATATGGATAAAAAACCACTAAACACTCTGATATCTGCAATCGGG
*Mm*PylRS_V31I_F	CATAAAATAAAACACCACGAAATTTCTCGAAGCAAAATC
*Mm*PylRS_V31I_R	CAATATAGATTTTGCTTCGAGAAATTTCGTGGTGTTT
*Mm*PylRS-I36V_F	CACCACGAAGTCTCTCGAAGCAAAGTCTATATTG

*Mm*PylRS-I36V_R	CATGCCATTTCAATATAGACTTTGCTTCGAGAGAC
*Mm*PylRS-T56P_F	CTTGTTGTAAACAACTCCAGGAGCAGCAGGCCCGCAAG
*Mm*PylRS-T56P_R	GAGCGCTCTTGCGGGCCTGCTGCTCCTGGAGTTGTTTAC
*Mm*PylRS-R61K_F	GCAGGACTGCAAGAGCGCTCAAACACCACAAATACAGGAAG
*Mm*PylRS-R61K_R	TTTGCAGGTCTTCCTGTATTTGTGGTGTTTGAGCGCTCTTG
*Mm*PylRS-H62Y_F	GCAGGACTGCAAGAGCGCTCAGGTATCACAAATACAGGAAG
*Mm*PylRS-H62Y_R	TTTGCAGGTCTTCCTGTATTTGTGATACCTGAGCGCTCTTG
*Mm*PylRS-H63Y_F	GCAGGACTGCAAGAGCGCTCAGGCACTATAAATACAGGAAG
*Mm*PylRS-H63Y_R	TTTGCAGGTCTTCCTGTATTTATAGTGCCTGAGCGCTCTTG
*Mm*PylRS-R61K/H63Y_F	GCAGGACTGCAAGAGCGCTCAAACACTATAAATACAGGAAG
*Mm*PylRS-R61K/H63Y_R	TTTGCAGGTCTTCCTGTATTTATAGTGTTTGAGCGCTCTTG
*Mm*PylRS_A100E_F	GCGTAAAAGTCAAGGTCGTTTCTGAGCCTACCAGAACG
*Mm*PylRS-A100E_R	GGCATTGCCTTTTTCGTTCTGGTAGGCTCAGAAACGACC
*Mm*PylRS-S193R_F	CAGGCAAGTGCCCCCGCACTTACGAAGCGTCAGACTGACAG
*Mm*PylRS-S193R_R	GACTTCAAGCCTGTCAGTCTGACGCTTCGTAAGTGCG
pBU16_co*Mm*PylRS_NdeI_F	AGAGGCATATGGATAAAAAACCACTAAACACTCTGATCTCTGCTACTG
pBU16_co*Mm*PylRS_PstI_R	GAAACTGCAGTTTCCATGGTTACAGGTTGGTAGAAATCCCGTTATAATAC
co*Mm*PylRS-N346S/C348M_F	CTGGAGGAGTTTACCATGCTGAGCTTTATGCAAATGG
co*Mm*PylRS-N346S/C348M_R	CAACCTGAACCCATTTGCATAAAGCTCAGCATGGTAAACTCC
co*Mm*PylRS-V401G_F	GCGACCTGGAACTGTCTAGTGCCGGCGTTGGACCAATTC
co*Mm*PylRS-V401G_R	GTCCAGCGGAATTGGTCCAACGCCGGCACTAGACAGTTCCAGG
co*Mm*PylRS-R61K/H63Y_F	GCTCTTCTCGTACAGCACGTGCACTGAAACACTATAAATATC
co*Mm*PylRS-R61K/H63Y_R	GTTTACAGGTTTTACGATATTTATAGTGTTTCAGTGCACGTG
co*Mm*PylRS-S193R_F	CAAGCATCAGCTCCAGCACTGACAAAACGTCAAACCGATCGTC
co*Mm*PylRS-S193R_R	GAACCTCCAGACGATCGGTTTGACGTTTTGTCAGTGCTGG

Supplementary Figure S1

The initial aaRS screening results indicated that the constructed *Mm*PylRS variants were capable of activating Trp analogs. The *E. coli* strain BL21(DE3) was cultured in M9 minimal medium (with Amp and Kan) and distributed to 200 μL in 96-wells plates containing different ncAAs (1 mM) in each well (see Table S2 and S3). The assay plates were incubated at 37 °C with shaking for 18 h. Fluorescence intensities and OD_{600} were measured, the former via bottom reading (excitation wavelength of 481 nm, emission wavelength of 511 nm).

A.

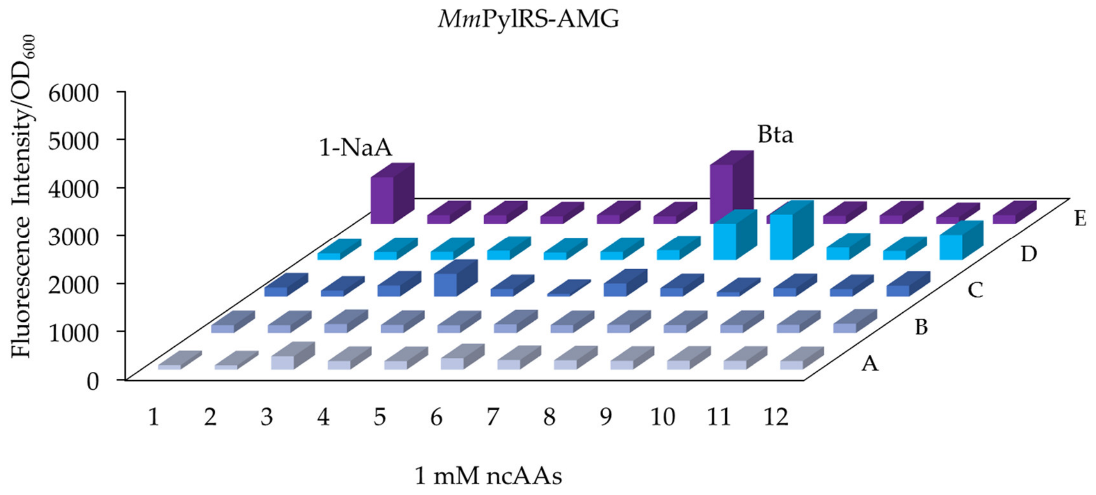

Substrate range of *Mm*PylRS-N346A/C348M/V401G.
The library of amino acids was tested for incorporation into sfGFP-R2TAG by *Mm*PylRS-AMG as measured via the reporter fluorescence intensity.

B.

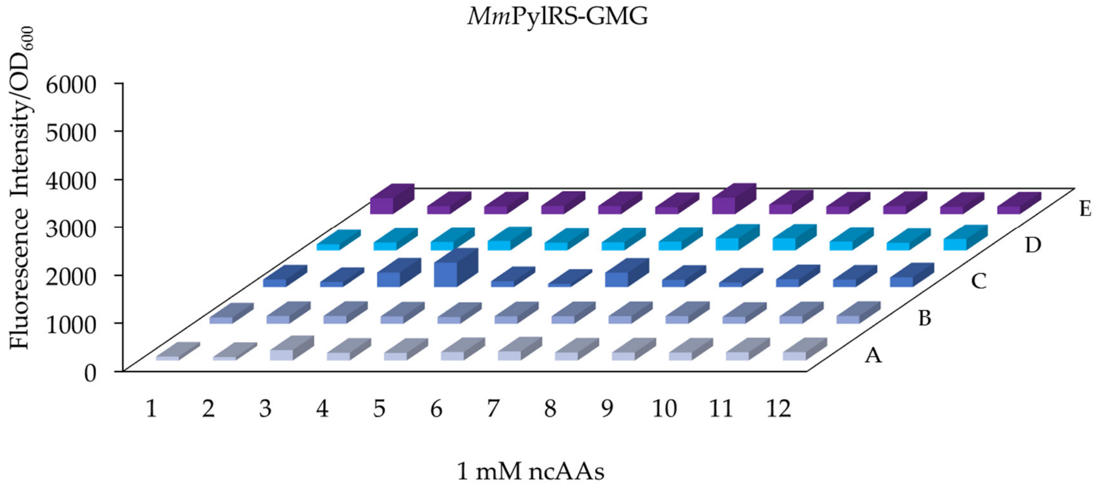

Substrate range of *Mm*PylRS-N346G/C348M/V401G.
The library of amino acids was tested for incorporation into sfGFP-R2TAG by *Mm*PylRS-GMG as measured via the reporter fluorescence intensity.

C.

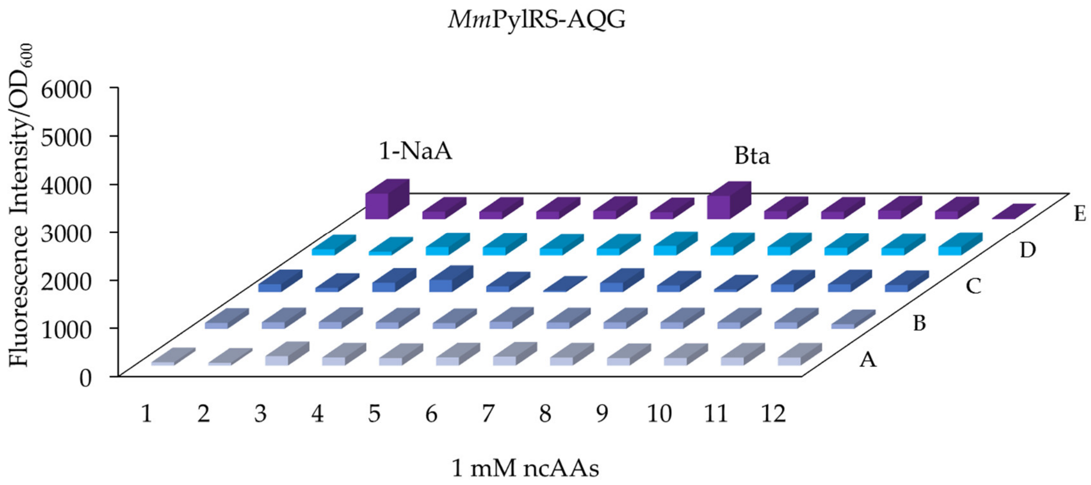

Substrate range of *Mm*PylRS-N346A/C348Q/V401G.
The library of amino acids was tested for incorporation into sfGFP-R2TAG by *Mm*PylRS-AQG as measured via the reporter fluorescence intensity.

D.

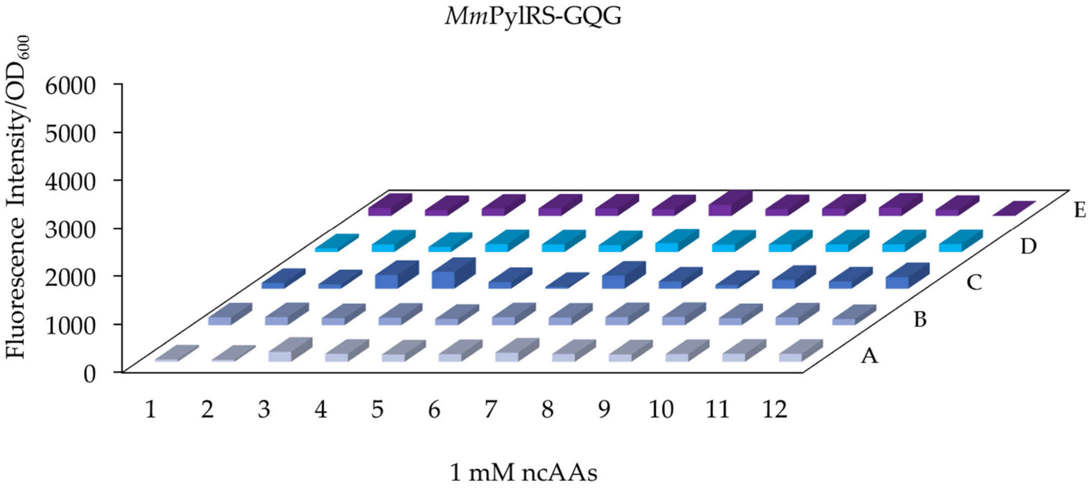

Substrate range of *Mm*PylRS-N346G/C348Q/V401G.
The library of amino acids was tested for incorporation into sfGFP-R2TAG by *Mm*PylRS-GQG as measured via the reporter fluorescence intensity.

E.

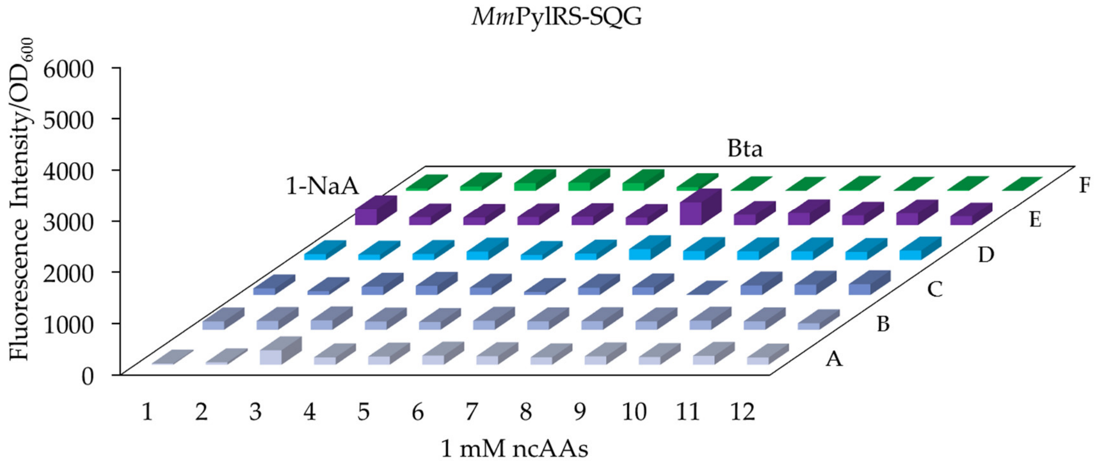

Substrate range of *Mm*PylRS-N346S/C348Q/V401G.
The library of amino acids was tested for incorporation into sfGFP-R2TAG by *Mm*PylRS-SQG as measured via the reporter fluorescence intensity.

F.

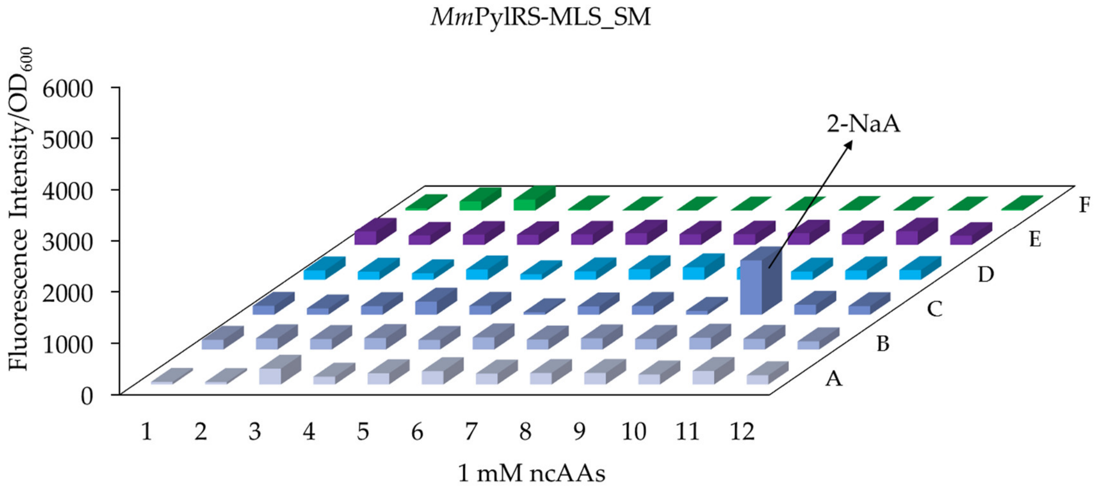

Substrate range of *Mm*PylRS- L305M/Y306L/L309S/N346S/C348M.
The library of amino acids was tested for incorporation into sfGFP-R2TAG by *Mm*PylRS-MLS_SM as measured via the reporter fluorescence intensity.

G.

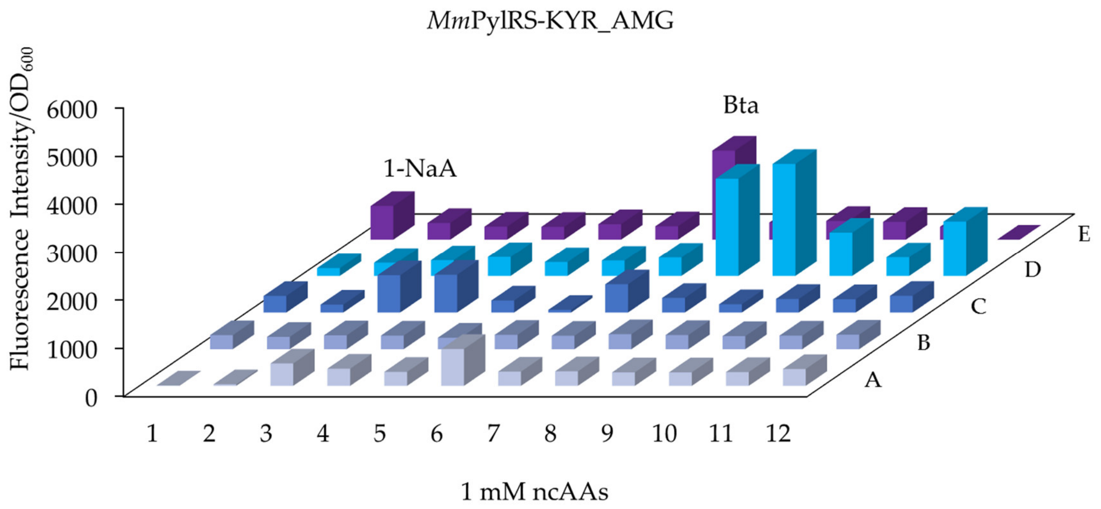

Substrate range of *Mm*PylRS-R61K/H63Y/S193R/N346A/C348M/V401G.
The library of amino acids was tested for incorporation into sfGFP-R2TAG by *Mm*PylRS-KYR_AMG as measured via the reporter fluorescence intensity.

H.

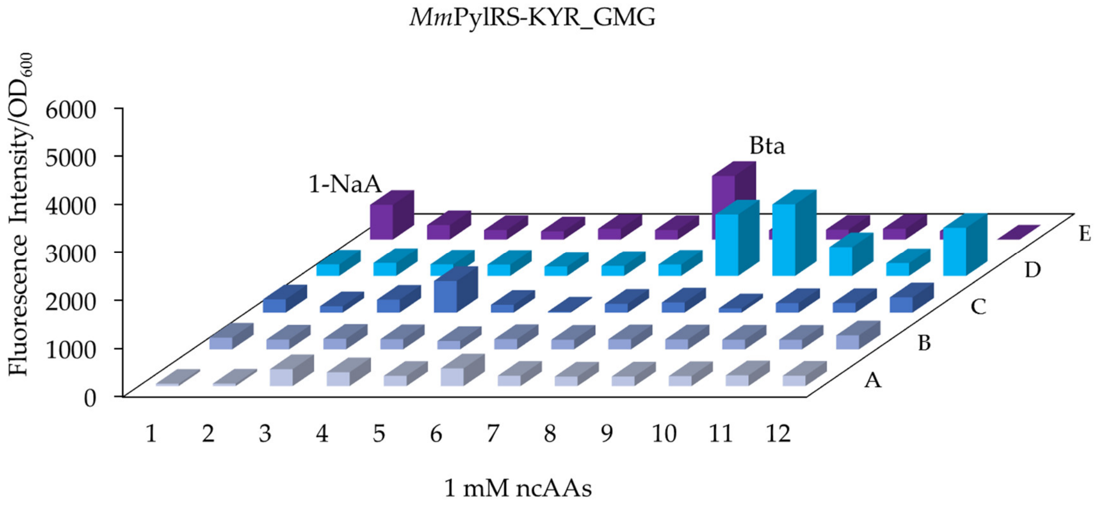

Substrate range of *Mm*PylRS-R61K/H63Y/S193R/N346G/C348M/V401G.
The library of amino acids was tested for incorporation into sfGFP-R2TAG by *Mm*PylRS-KYR_GMG as measured via the reporter fluorescence intensity.

I.

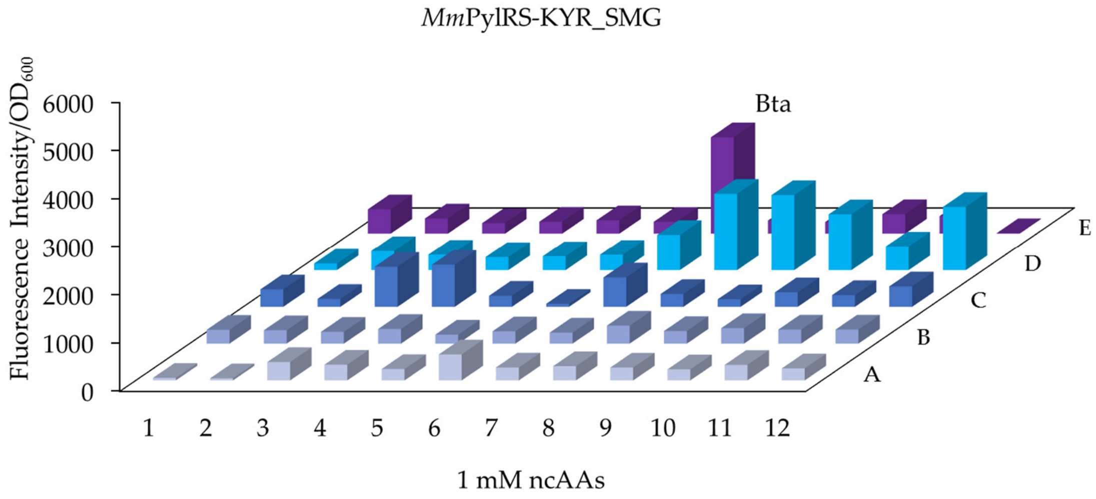

Substrate range of *Mm*PylRS-R61K/H63Y/S193R/N346S/C348M/V401G.
The library of amino acids was tested for incorporation into sfGFP-R2TAG by *Mm*PylRS-KYR_SMG as measured via the reporter fluorescence intensity.

J.

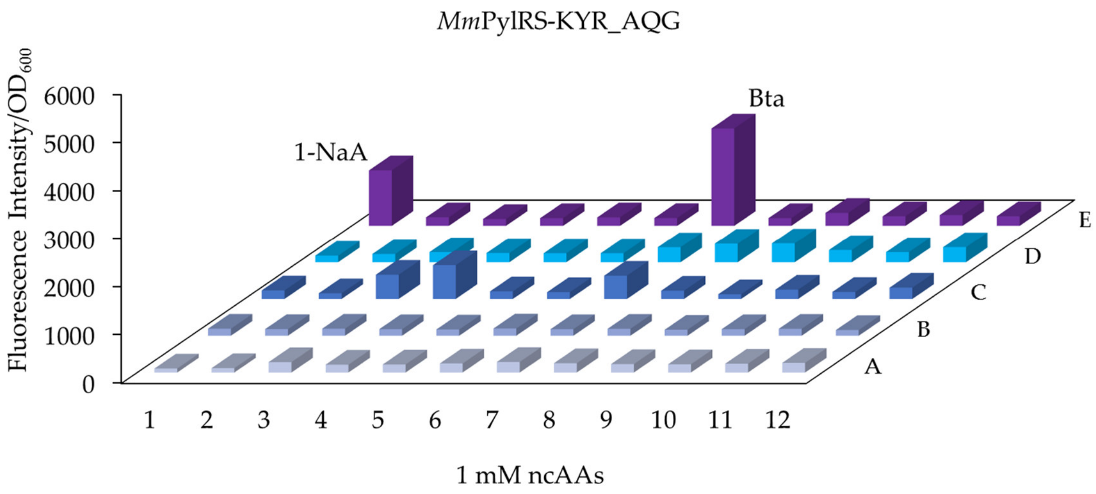

Substrate range of *Mm*PylRS-R61K/H63Y/S193R/N346A/C348Q/V401G.
The library of amino acids was tested for incorporation into sfGFP-R2TAG by *Mm*PylRS-KYR_AQG as measured via the reporter fluorescence intensity.

K.

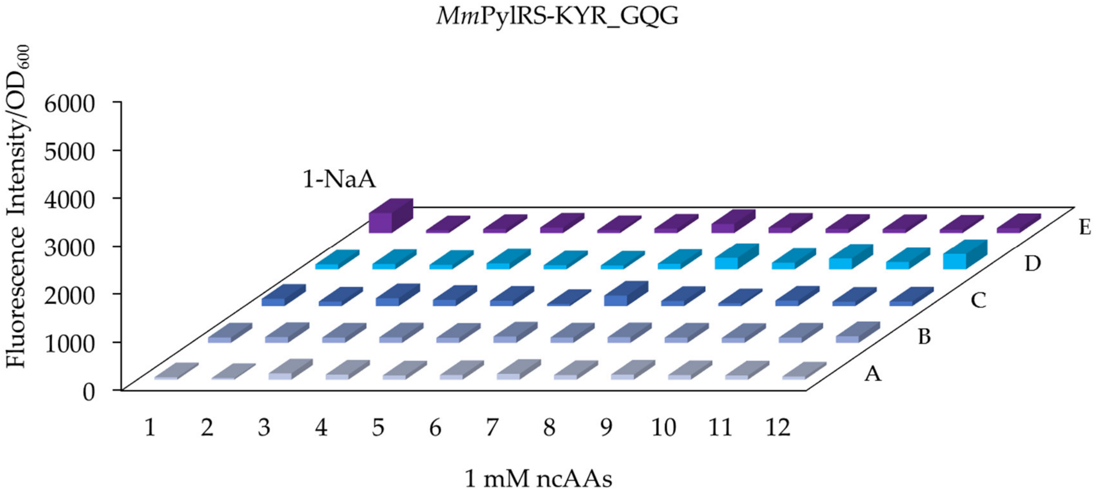

Substrate range of *Mm*PylRS-R61K/H63Y/S193R/N346G/C348Q/V401G.
The library of amino acids was tested for incorporation into sfGFP-R2TAG by *Mm*PylRS-KYR_GQG as measured via the reporter fluorescence intensity.

L.

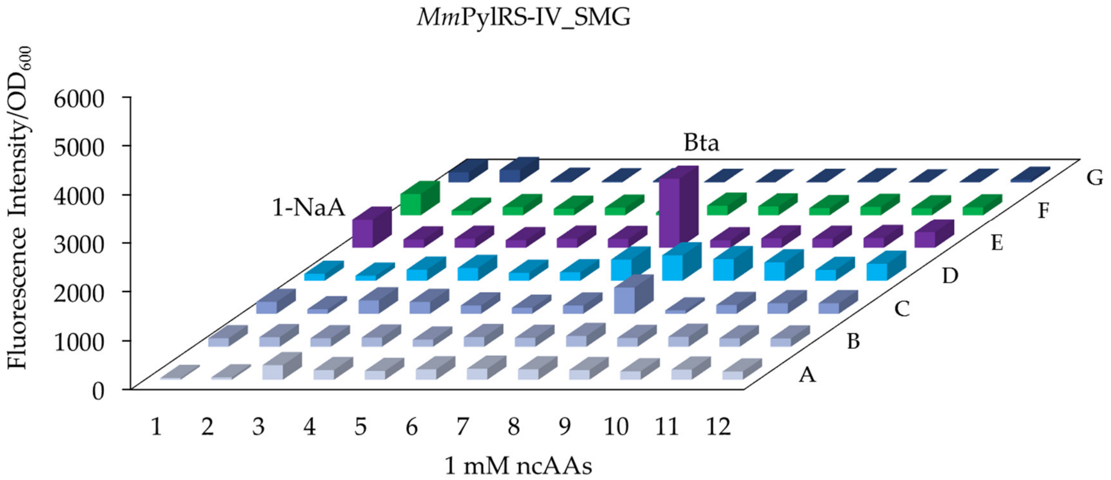

Substrate range of *Mm*PylRS-T13I/I36V/N346S/C348M/V401G.
The library of amino acids was tested for incorporation into sfGFP-R2TAG by *Mm*PylRS-IV_SMG as measured via the reporter fluorescence intensity.

M.

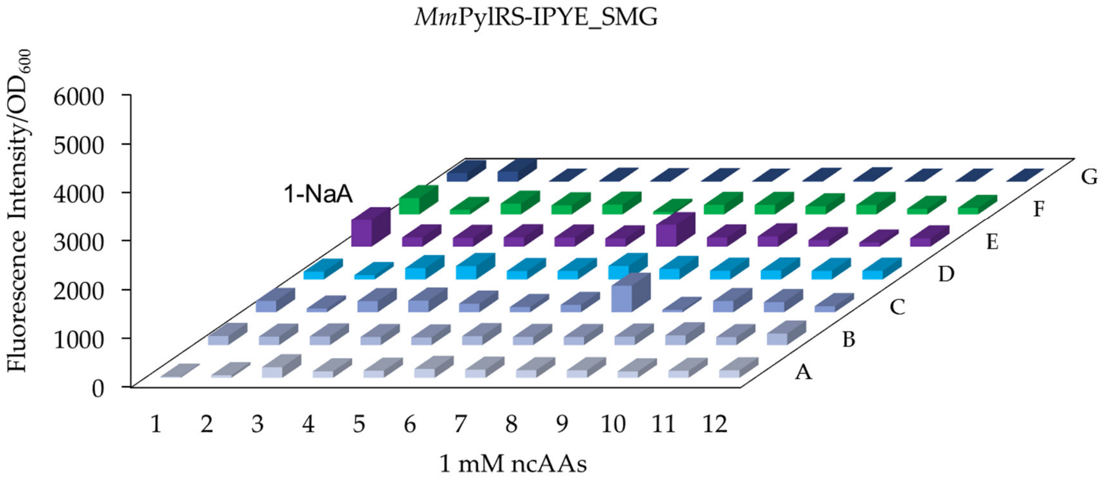

Substrate range of *Mm*PylRS-V31I/T56P/H62Y/A100E/N346S/C348M/V401G.
The library of amino acids was tested for incorporation into sfGFP-R2TAG by *Mm*PylRS-IPYE_SMG as measured via the reporter fluorescence intensity.

N.

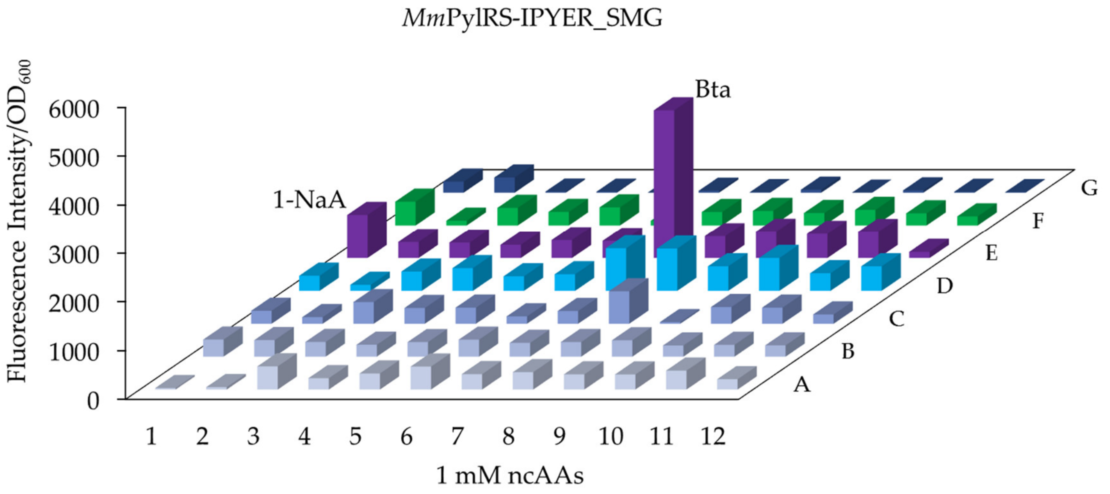

Substrate range of *Mm*PylRS-V31I/T56P/H62Y/A100E/S193R/N346S/C348M/V401G.
The library of amino acids was tested for incorporation into sfGFP-R2TAG by *Mm*PylRS-IPYER_SMG as measured via the reporter fluorescence intensity.

O.

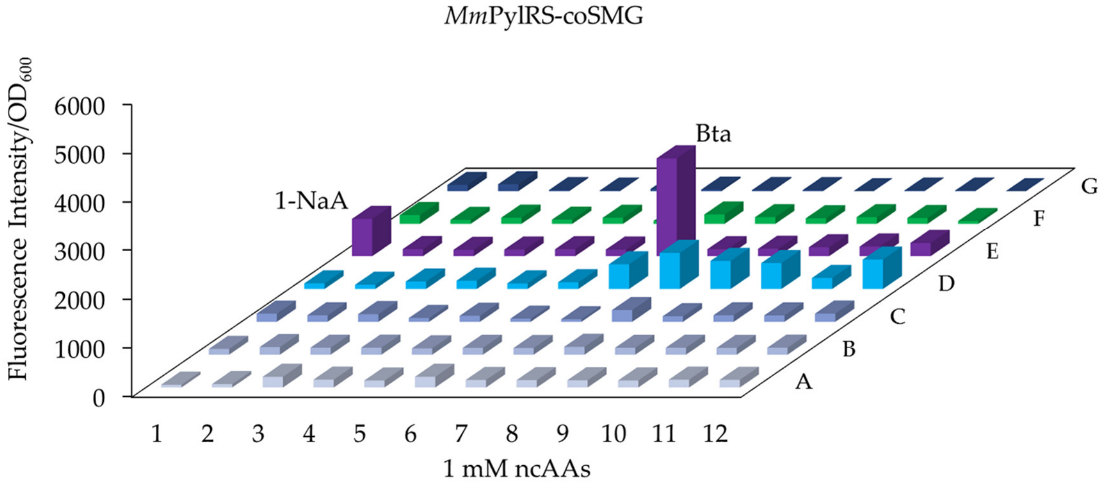

Substrate range of codon optimized *Mm*PylRS-N346S/C348M/V401G.
The library of amino acids was tested for incorporation into sfGFP-R2TAG by *Mm*PylRS-coSMG as measured via the reporter fluorescence intensity.

Supplementary Figure S2

Reporter-based aaRS screening results indicating that mutations of PylRS residue W417 can lead to activation of Tyr analogs. The *E. coli* strain BL21(DE3) was cultured in M9 minimal medium (with Amp and Kan) and distributed to 200 µL in 96-wells plates containing different ncAAs 1 mM in each well (see Table S2 and S3). The assay plates were incubated with shaking for 18 h at 37 °C. Fluorescence intensities and OD_{600} were measured, the former via bottom reading (excitation wavelength of 481 nm, emission wavelength of 511 nm).

A.

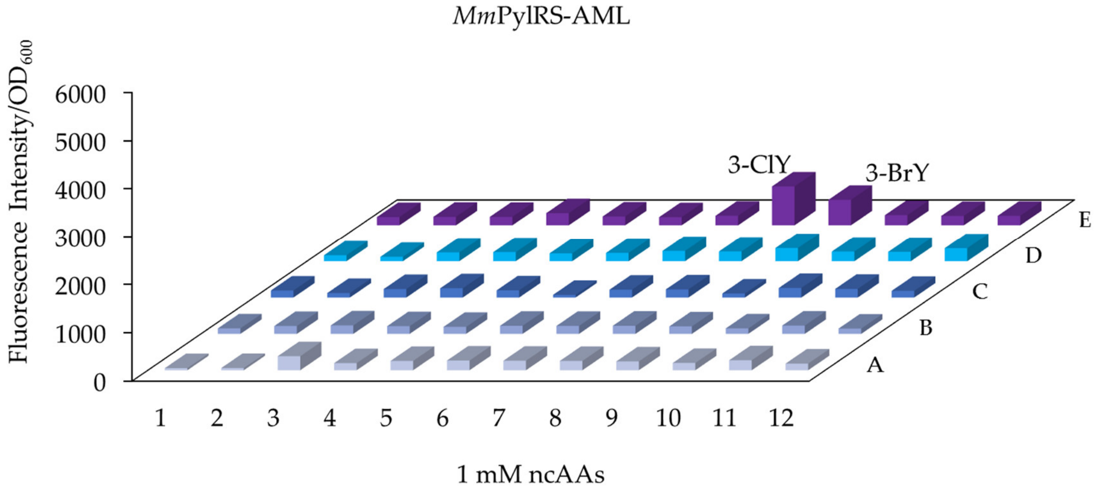

Substrate range of *Mm*PylRS-N346A/C348M/W417L.
The library of amino acids was tested for incorporation into sfGFP-R2TAG by *Mm*PylRS-AML as measured via the reporter fluorescence intensity.

B.

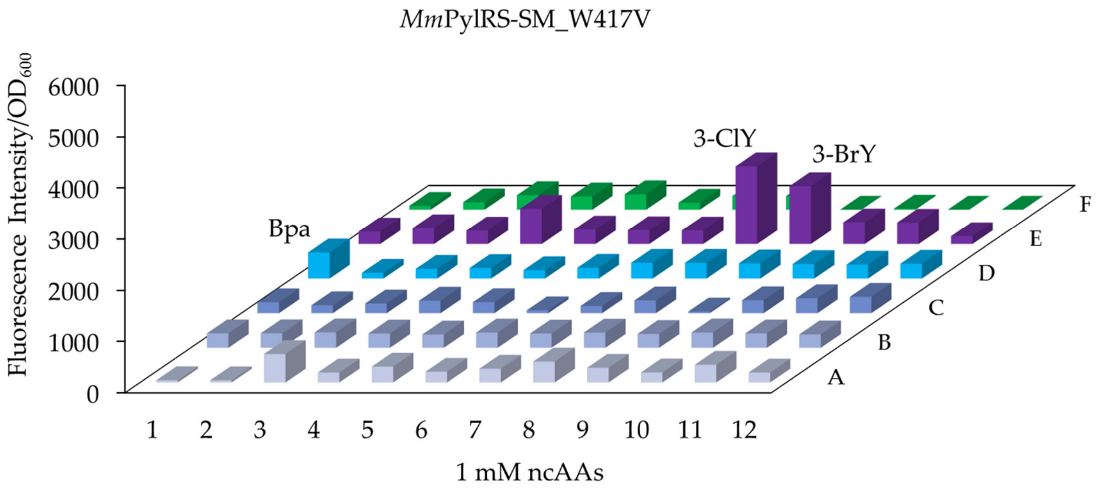

Substrate range of *Mm*PylRS-N346S/C348M/W417V.
The library of amino acids was tested for incorporation into sfGFP-R2TAG by *Mm*PylRS-SM_W417V as measured via the reporter fluorescence intensity.

C.

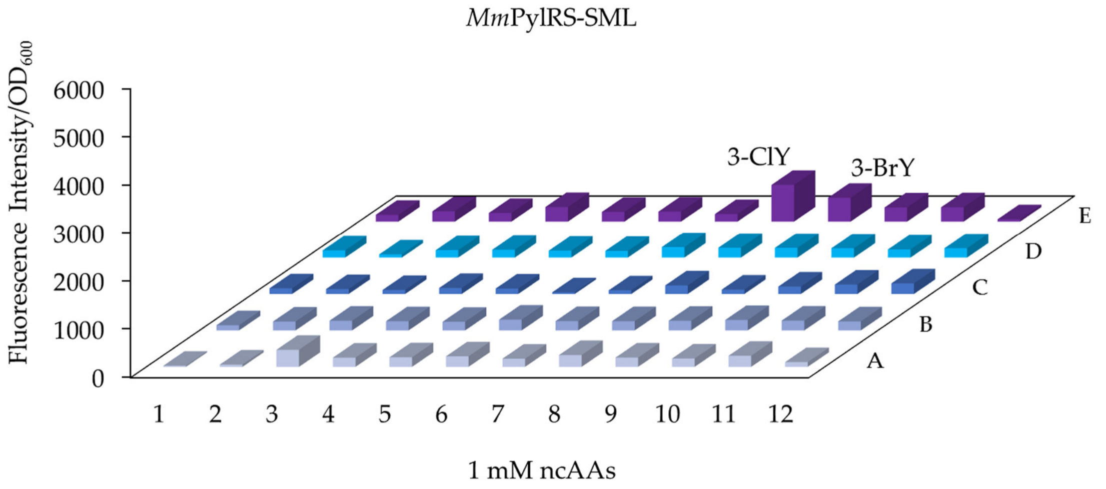

Substrate range of *Mm*PylRS-N346S/C348M/W417L.
The library of amino acids was tested for incorporation into sfGFP-R2TAG by *Mm*PylRS-SML as measured via the reporter fluorescence intensity.

D.

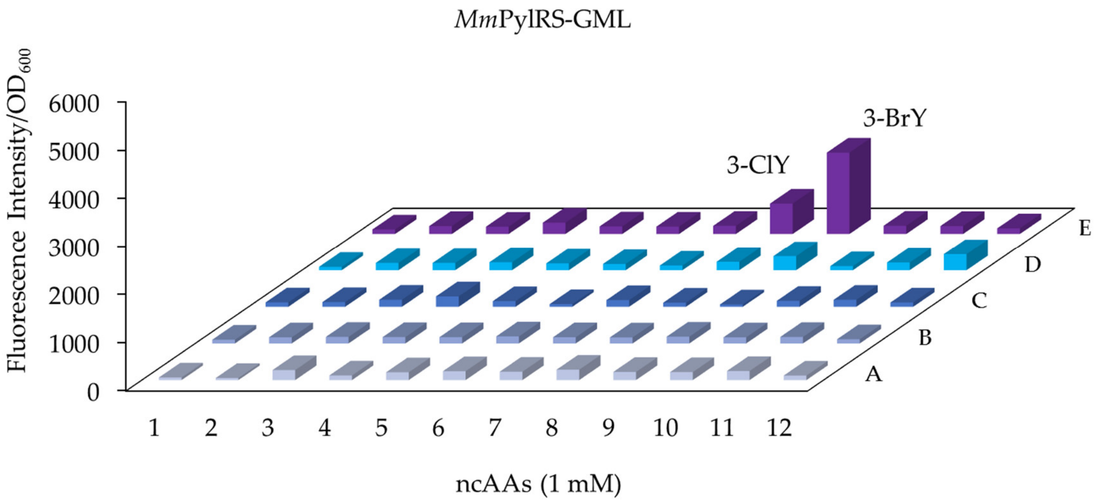

Substrate range of *Mm*PylRS-N346G/C348M/W417L.

The library of amino acids was tested for incorporation into sfGFP-R2TAG by *Mm*PylRS-GML as measured via the reporter fluorescence intensity.

E.

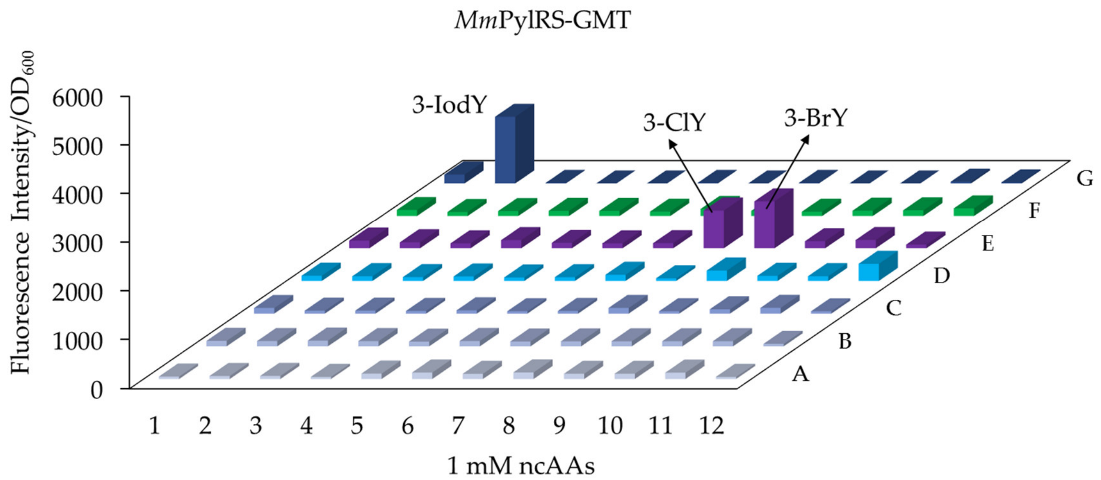

Substrate range of *Mm*PylRS-N346G/C348M/W417T.

The library of amino acids was tested for incorporation into sfGFP-R2TAG by *Mm*PylRS-GMT as measured via the reporter fluorescence intensity.

F.

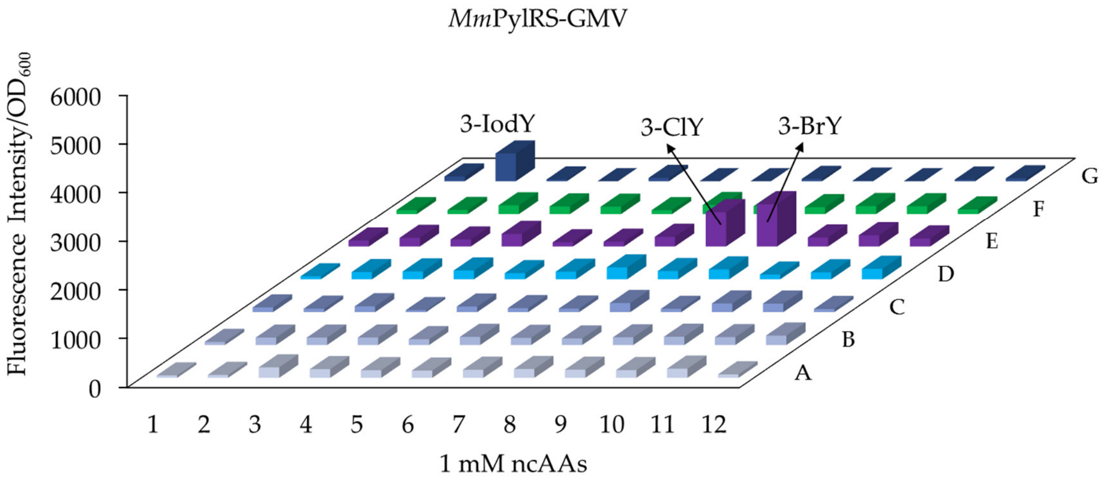

Substrate range of *Mm*PylRS-N346G/C348M/W417V.

The library of amino acids was tested for incorporation into sfGFP-R2TAG by *Mm*PylRS-GMV as measured via the reporter fluorescence intensity.

G.

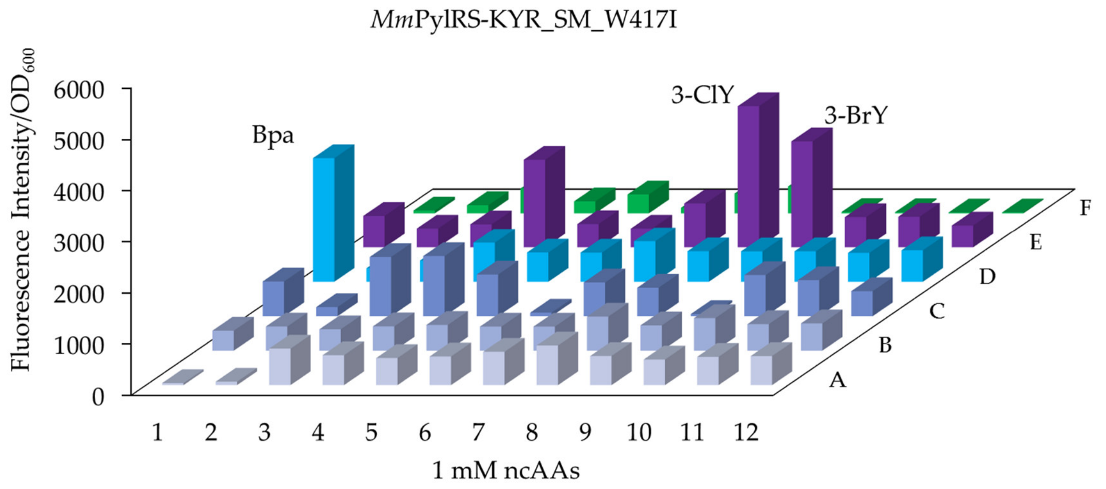

Substrate range of *Mm*PylRS-R61K/H63Y/S193R/N346S/C348M/W417I.

The library of amino acids was tested for incorporation into sfGFP-R2TAG by *Mm*PylRS-KYR_SM_W417I as measured via the reporter fluorescence intensity.

H.

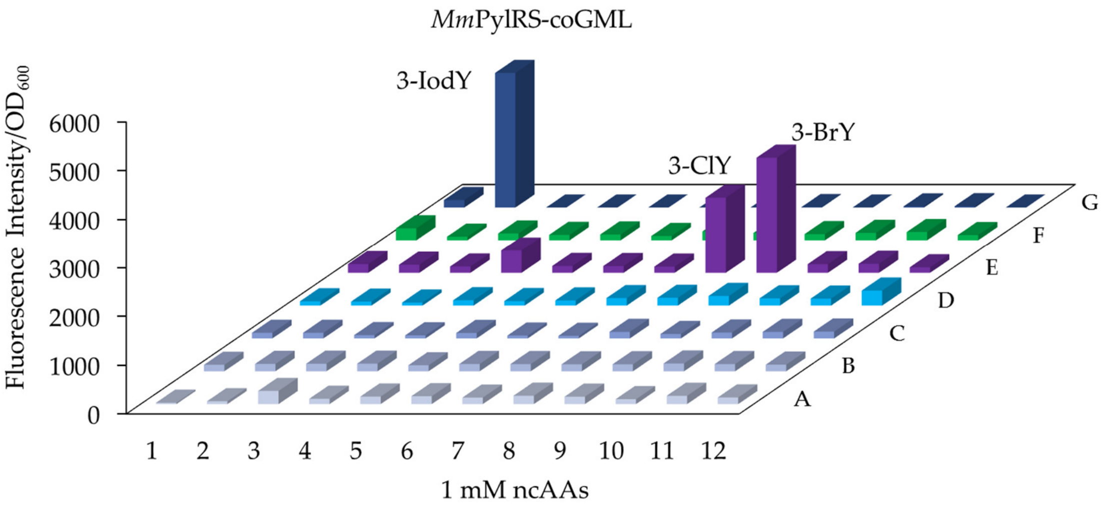

Substrate range of codon optimized *Mm*PylRS-N346G/C348M/W417L.
The library of amino acids was tested for incorporation into sfGFP-R2TAG by *Mm*PylRS-coGML as measured via the reporter fluorescence intensity.

Supplementary Figure S3

Comparison of Bta incorporation efficiency mediated by screened *Mm*PylRS variants. Fluorescence was generated through suppression of the amber stop codon located in the reporter gene (sfGFP-R2TAG) by different *Mm*PylRS variants through ribosomal incorporation of Bta. *Mm*PylRS-SMG showed higher fluorescence intensity than other synthetase variants. Control setup is without induction and ncAA supplementation; no ncAA: control with IPTG induction but without Trp or Bta supplementation, respectively. Trp supplementation did not lead to pronounced signal changes (cf. no ncAA samples). Data are means ± SD (n = 3).

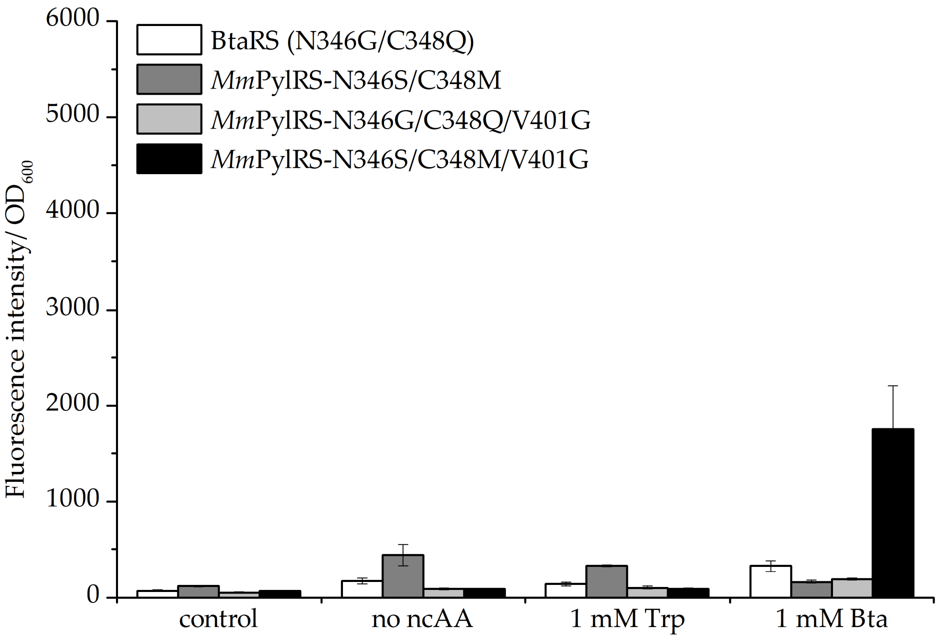

Supplementary Figure S4

Comparison Bta incorporation efficiency between codon-optimized *Mm*PylRS-coSMG (coSMG) and unoptimized *Mm*PylRS-SMG (SMG). Intact cell fluorescence was generated through suppression of the amber stop codon in the sfGFP-R2TAG reporter gene. Different *Mm*PylRS setups were tested for Trp and Bta incorporation. Control setup is without induction and ncAA supplementation; no ncAAs: control with IPTG induction but without Trp or Bta supplementation, respectively. Data are means ± SD (n = 3).

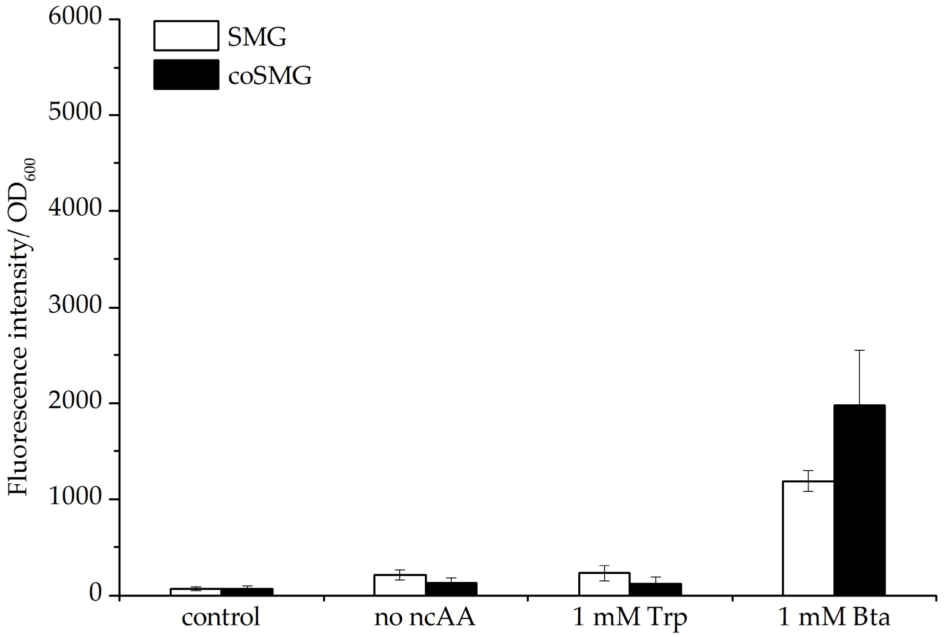

Supplementary Figure S5

Comparison of Bta incorporation efficiency by *Mm*PylRS-SMG variants with mutagenesis of additional aaRS positions (T13, V31, I36, T56, R61, H62, H63, A100, and S193). Intact cell fluorescence was generated through suppression of the amber stop codon in the sfGFP-R2TAG reporter gene. Different *Mm*PylRS setups were tested for Trp or Bta incorporation. Control setup is without induction and ncAA supplementation; no ncAAs: control with IPTG induction but without Trp or Bta supplementation, respectively. Data are means ± SD (n = 3).

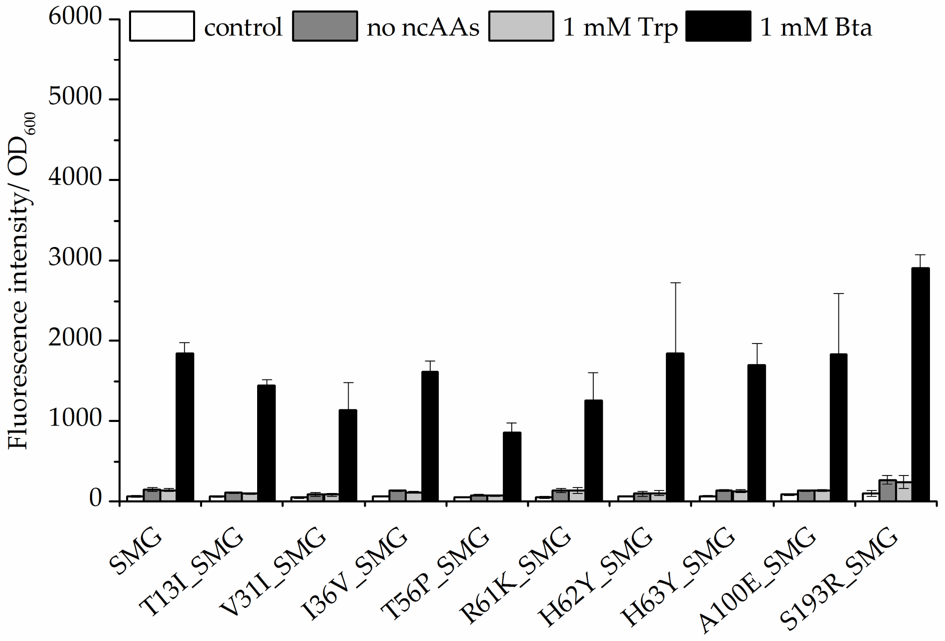

Supplementary Figure S6

Comparison of Bta incorporation efficiency by *Mm*PylRS-SMG co-expression upon full mutagenesis of aaRS position S193. Intact cell fluorescence was generated through suppression of the amber stop codon in the sfGFP-R2TAG reporter gene. Different *Mm*PylRS variants were tested for Trp or Bta incorporation. Control setup is without induction and ncAA supplementation; no ncAAs: control with IPTG induction but without amino acids supplementation. Data are means ± SD (n = 3).

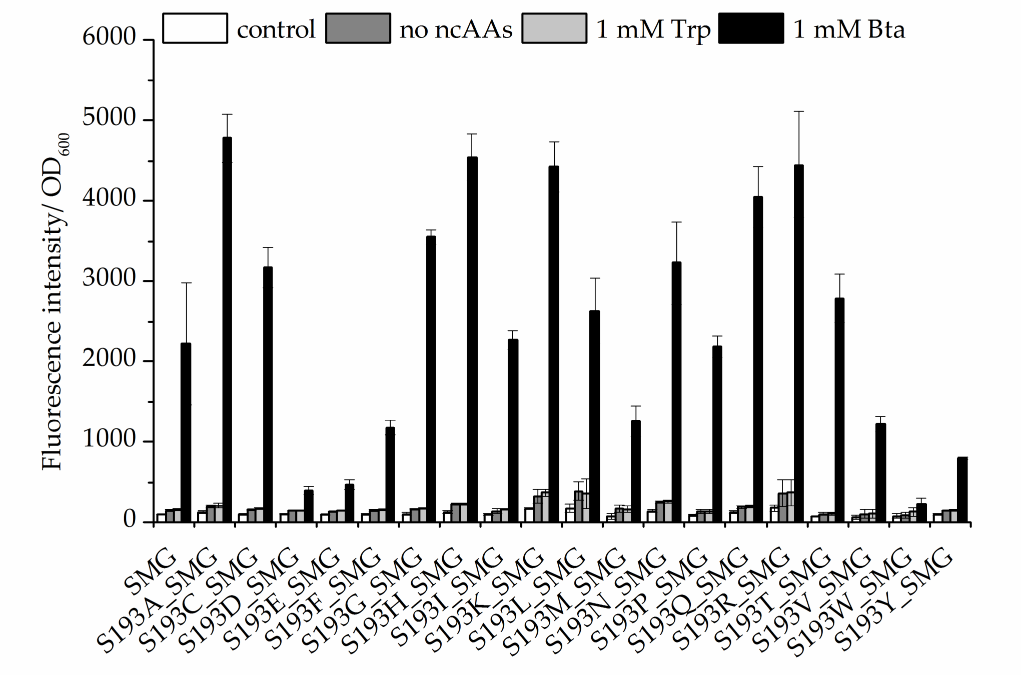

Supplementary Figure S7

Comparison of Bta incorporation efficiency by *Mm*PylRS-SMG variants with mutagenesis of additional aaRS positions (T13, V31, I36, T56, R61, H62, H63, and A100) combined with S193R replacement. Intact cell fluorescence was generated through suppression of the amber stop codon in the sfGFP-R2TAG reporter gene. Different *Mm*PylRS variants were tested for Trp or Bta incorporation. Control setup is without induction and ncAA supplementation; no ncAAs: control with IPTG induction but without Trp or Bta supplementation, respectively. Data are means ± SD (n = 3).

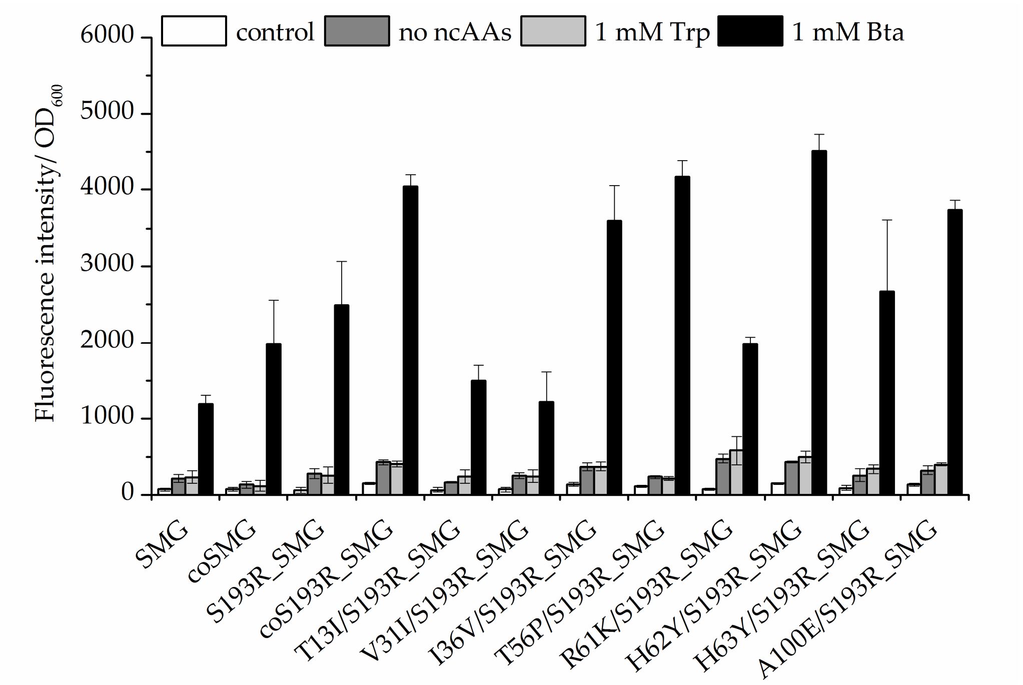

Supplementary Figure S8

SDS-PAGE of SUMO-sfGFP (R2TAG) expression in presence of 1 mM Bta with co-expression of *Mm*PylRS-SMG, *Mm*PylRS-coSMG (codon-optimized gene variant), *Mm*PylRS-KYR_SMG, or *Mm*PylRS-13IPYER_SMG, respectively. (M: protein ladder; 1: whole cell extract non-induced; 2: whole cell extract with 1 mM IPTG induction and 1 mM Bta; 3: cell lysate; 4: affinity column flow-through; 5: wash fraction with 20 mM imidazole in PBS buffer; 6: elution fraction with 300 mM imidazole in PBS buffer). (FL = full-length, TR= truncated product).

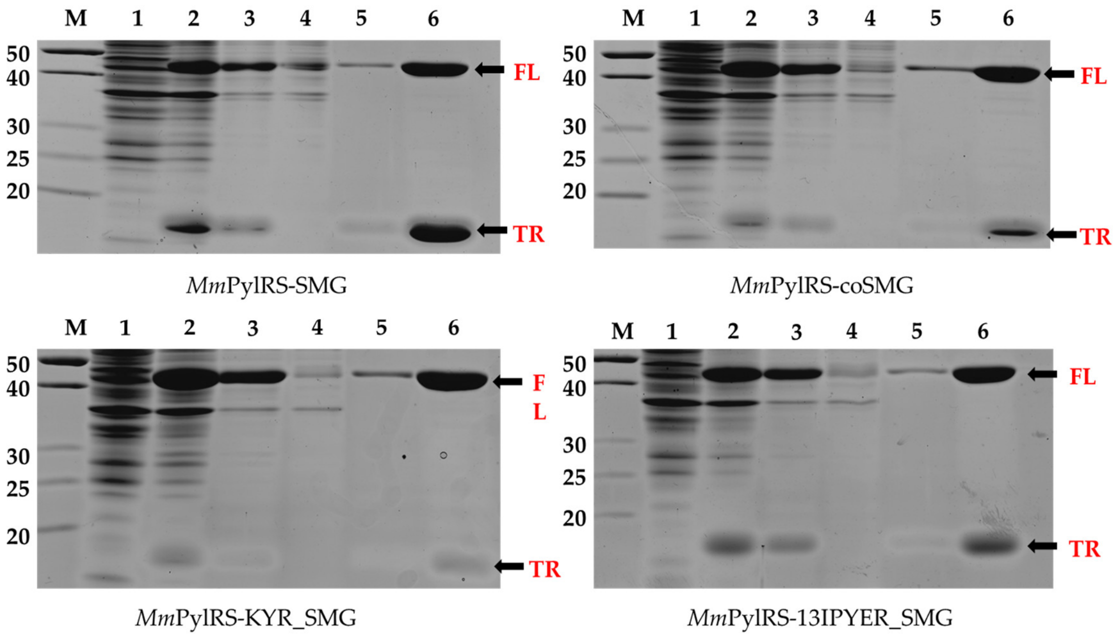

Supplementary Figure S9

Deconvoluted ESI-MS spectra of purified sfGFP modified by site-specific incorporation of ncAAs. Observed and calculated intact protein masses are as follows: sfGFP-R2 3-MeY calculated is 27774.3 Da, observed is 27774 Da; sfGFP-R2 1-MeW calculated is 27797.3 Da, observed are 27726 Da (background signal) and 27796 Da; sfGFP-R2 AzAla calculated is 27794.3 Da, observed are 27725 Da (background signal) and 27794 Da; sfGFP-R2 2-NaA calculated is 27794.3 Da, observed is 27794 Da; sfGFP-R2 Bpa calculated is 27848.3 Da, observed is 27848 Da. Background signal: The theoretical mass of sfGFP with glutamine (Gln) incorporation is 27725 Da.)

Commonly referred to as near-cognate suppression, Gln incorporation is known to occur naturally in *E. coli* at amber stop codon sites, with the endogenous $tRNA^{Gln}_{CUG}$ recognizing the UAG codon [1–4]).

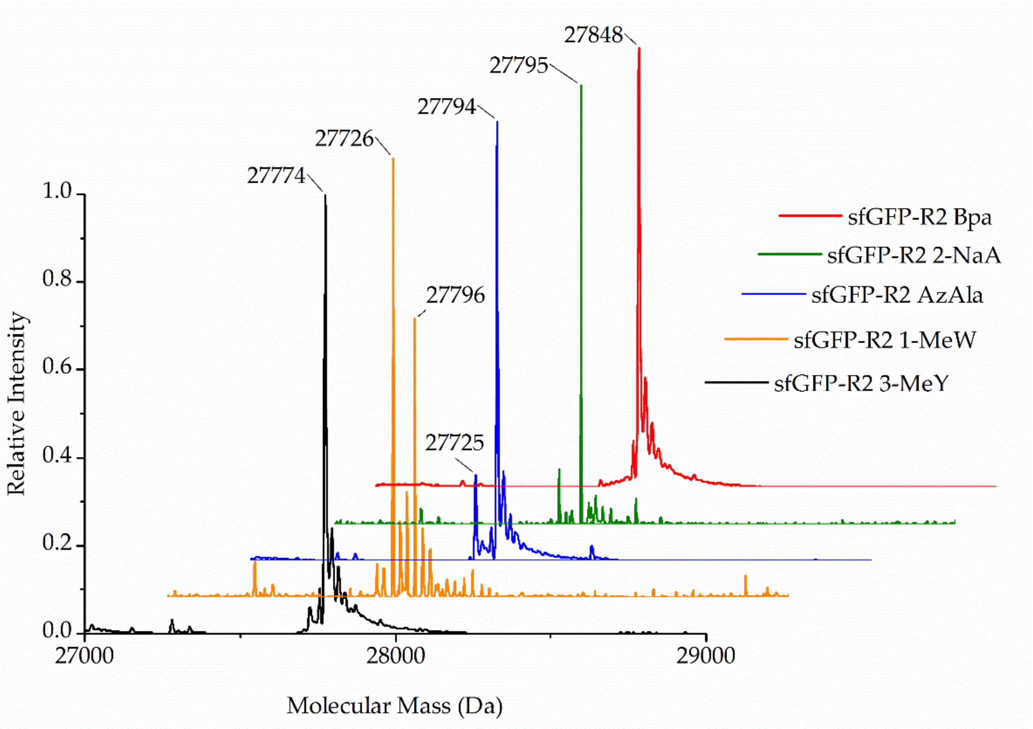

Supplementary Figure S10

Comparison between the expression of wild-type (amber codon free) sfGFP and the suppression of the amber stop codon at sfGFP position 2 (sfGFP-R2TAG) by screened *Mm*PylRS variants. Fluorescence was generated by reporter gene (wt-sfGFP) expression as well as by suppression of the amber stop codon located in the reporter gene (sfGFP-R2TAG) mediated by different *Mm*PylRS variants through ribosomal incorporation of Bta. Control setup is without induction, AAs and ncAAs supplementation; no ncAAs: control with IPTG induction but without Trp or Bta supplementation, respectively. Data are means ± SD (n = 3).

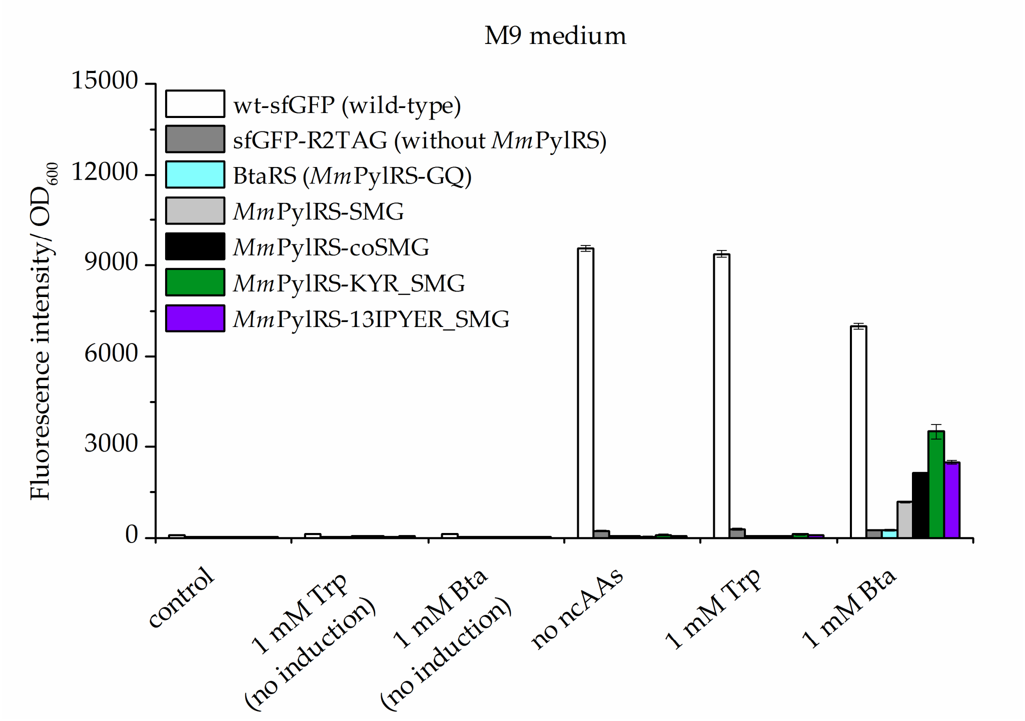

Supplementary Figure S11

Comparing the expression of wt-sfGFP and the suppression of the amber stop codon in sfGFP (sfGFP-R2TAG) by Bta incorporation via screened *Mm*PylRS variants in different growth media. The setup of the sfGFP-R2TAG controls lacks the o-pair plasmid. Data are means ± SD (n = 3).

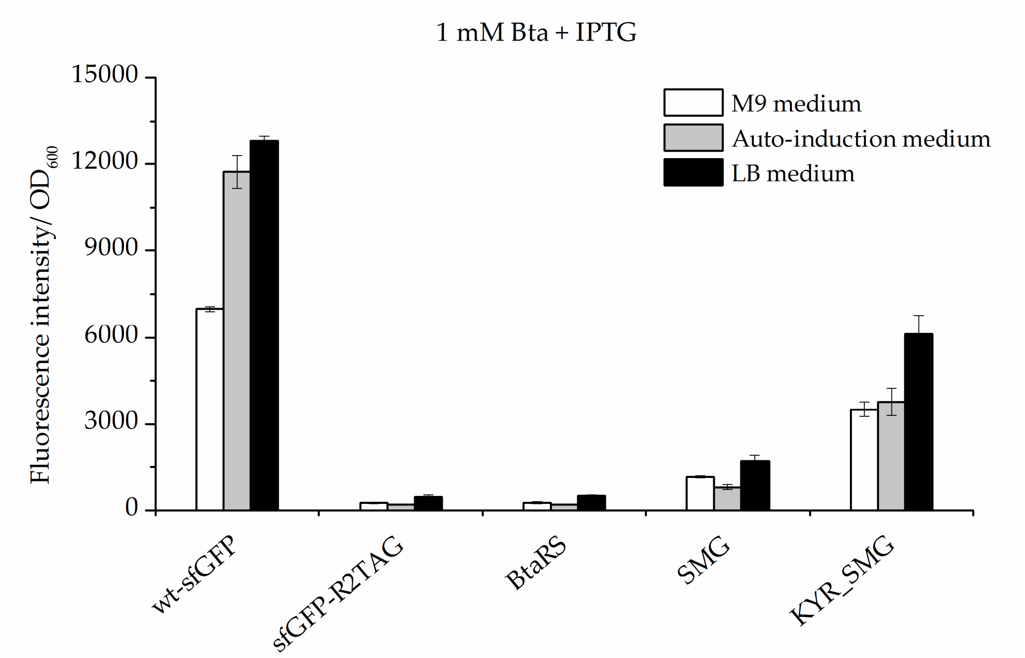

* Auto-induction medium: 0.5% glycerol, 0.075% glucose, 0.05% a-Lactose monohydrate, 2 mM MgSO4, 0.45% Monosodium succinate (pH 6.8), 25 mM Na_2HPO_4, 25 mM KH_2PO_4, 50 mM NH_4Cl, 5 mM Na_2SO_4, trace metals (10 µM $FeCl_3$, 4 µM $CaCl_2$, 2 µM $MnCl_2$, 2 µM $ZnSO_4$, 0.4 µM $CoCl_2$, 0.4 µM $CuCl_2$, 0.4 µM $NiCl_2$, 0.4 µM Na_2MoO_4, 0.4 µM Na_2SeO_3, 0.4 µM H_3BO_3), 0.2 mg/mL amino acids (each) [5].

* LB medium: 10 g/L tryptone, 5 g/L yeast extract, and 10 g/L NaCl.

Supplementary Figure S12

Deconvoluted ESI-MS spectra of purified sfGFP modified by site-specific incorporation of Bta.

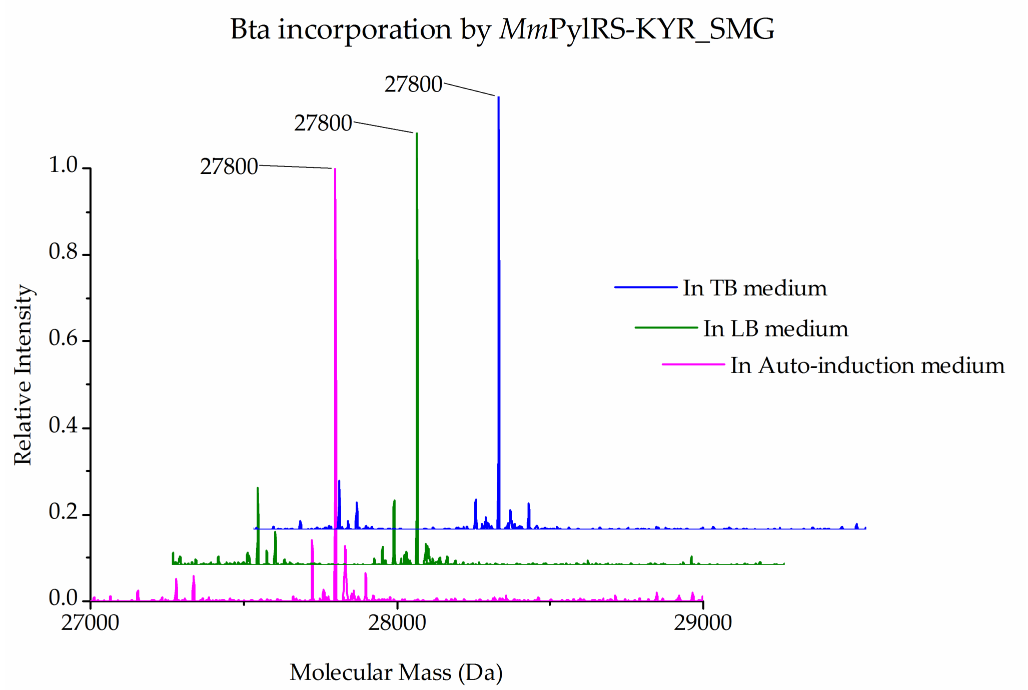

A. Deconvoluted ESI-MS spectra of purified sfGFP-R2_Bta produced by *Mm*PylRS-KYR_SMG variant co-expression in rich media. The calculated molecular mass is 27800 Da whereas the observed mass is 27800 Da.

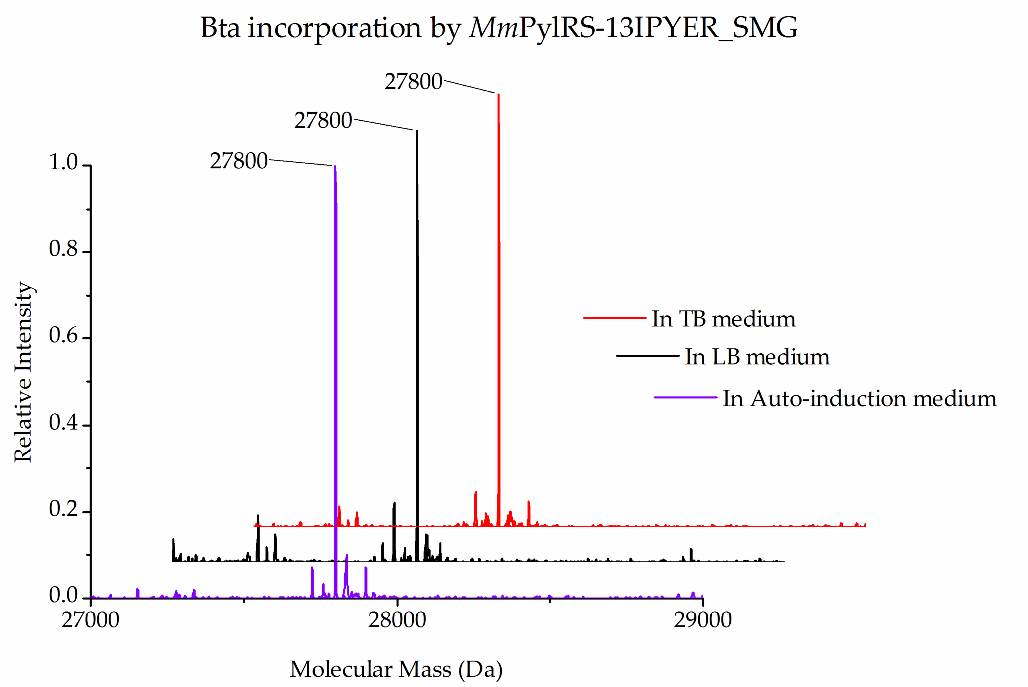

B. Deconvoluted ESI-MS spectra of purified sfGFP-R2_Bta produced by *Mm*PylRS-13IPYER_SMG variant co-expression in rich media. The calculated molecular mass is 27800 Da whereas the observed mass is 27800 Da.

Supplementary Table 1

Information on *Mm*PylRS variants used in this study. The usage of codon-optimized genes for aaRS co-expression is indicated by "co". Please note the scheme used to discriminate between the two Ile mutations at aaRS position 13 and 36, respectively.

Name	Mutations
*Mm*PylRS-SM	N346S/C348M
*Mm*PylRS-GQ	N346G/C348Q
*Mm*PylRS-GM	N346G/C348M
*Mm*PylRS-SMG	N346S/C348M/V401G
*Mm*PylRS-GQG	N346G/C348Q/V401G
*Mm*PylRS-GML	N346G/C348M/W417L
*Mm*PylRS-MLA_GQ	L305M/Y306L/L309A/N346G/C348Q
*Mm*PylRS-MLS_SM	L305M/Y306L/L309S/N346S/C348M
*Mm*PylRS-IV_SMG	T13I/I36V/N346S/C348M/V401G
*Mm*PylRS-IPYE_SMG	V31I/T56P/H62Y/A100E/N346S/C348M/V401G
*Mm*PylRS-KYR_SMG	R61K/H63Y/S193R/N346S/C348M/V401G
*Mm*PylRS-IVR_SMG	T13I/I36V/S193R/N346S/C348M/V401G
*Mm*PylRS-IPYER_SMG	V31I/T56P/H62Y/A100E/S193R/N346S/C348M/V401G
*Mm*PylRS-13IPYE_SMG	T13I/T56P/H62Y/A100E/N346S/C348M/V401G
*Mm*PylRS-13IPYER_SMG	T13I/T56P/H62Y/A100E/S193R/N346S/C348M/V401G
*Mm*PylRS-IIPYE_SMG	T13I/V31I/T56P/H62Y/A100E/N346S/C348M/ V401G
*Mm*PylRS-IIPYER_SMG	T13I/V31I/T56P/H62Y/A100E/S193R/N346S/ C348M/V401G
*Mm*PylRS-SMGL	N346S/C348M/V401G/W417L
*Mm*PylRS-SMGK	N346S/C348M/V401G/W417K
*Mm*PylRS-SMGI	N346S/C348M/V401G/W417I
*Mm*PylRS-KYR_SMGL	R61K/H63Y/S193R/N346S/C348M/V401G/ W417L
*Mm*PylRS-KYR_SMGK	R61K/H63Y/S193R/N346S/C348M/V401G/ W417K
*Mm*PylRS-KYR_SMGI	R61K/H63Y/S193R/N346S/C348M/V401G/ W417I
*Mm*PylRS-SML	N346S/C348M/W417L
*Mm*PylRS-SMV	N346S/C348M/W417V
*Mm*PylRS-KYR_SML	R61K/H63Y/S193R/N346S/C348M/W417L
*Mm*PylRS-KYR_SMI	R61K/H63Y/S193R/N346S/C348M/W417I
*Mm*PylRS-coSMG	N346S/C348M/V401G (codon-optimized)
*Mm*PylRS-coS193R_SMG	S193R/N346S/C348M/V401G (codon-optimized)
*Mm*PylRS-coKYR_SMG	R61K/H63Y/S193R/N346S/C348M/V401G (codon-optimized)

Supplementary Table 2

Layout of the amino acids screening system used for *Mm*PylRS variants in 96-well plate format.

	**1**	**2**	**3**	**4**	**5**	**6**	**7**	**8**	**9**	**10**	**11**	**12**
**A**	W/O	W/O	Met	Ala	Gly	Phe	Trp	Tyr	Val	Cys	His	Pro
**B**	No AAs	No AAs	Leu	Ile	Lys	Arg	Ser	Thr	Asn	Gln	Glu	Asp
**C**	1	2	3	4	5	6	7	8	9	10	11	12
**D**	13	14	15	16	17	18	19	20	21	22	23	24
**E**	25	26	27	28	29	30	31	32	33	34	35	36
**F**	37	38	39	40	41	42	43	44	45	46	47	48
**G**	49	50										
**H**												

- W/O: control without AAs, ncAAs, and IPTG induction; No AAs: control with IPTG induction but without amino acids supplementation.

Supplementary Table 3

Non-canonical amino acids (ncAAs) used in this study.

No.	Name	CAS No.	Company	Structure
1	5-Cyano-*DL*-Tryptophan (5-CNW)	2089602-82-2	Biosynth	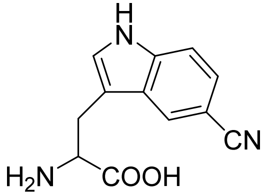
2	5-Hydroxy-*L*-Tryptophan (5-OHW)	145224-90-4	Biosynth	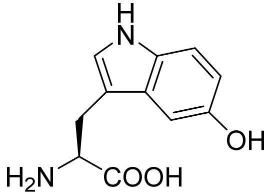
3	5-Bromo-*DL*-Tryptophan (5-BrW)	6548-09-0	Biosynth	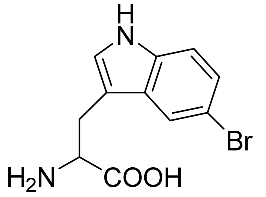
4	6-Bromo-*DL*-Tryptophan (6-BrW)	33599-61-0	Biosynth	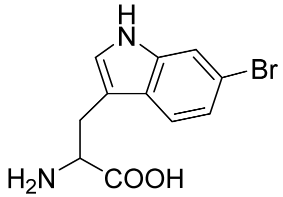
5	7-Bromo-*DL*-Tryptophan (7-BrW)	852391-45-8	Biosynth	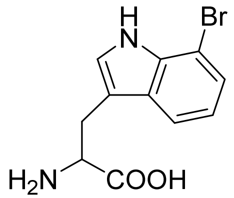

6	4-Chloro-*L*-Tryptophan (4-ClW)	52448-14-3	Biosynth	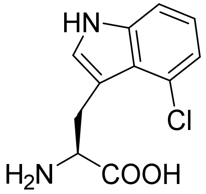
7	5-Chloro-*DL*-Tryptophan (5-ClW)	154-07-4	Biosynth	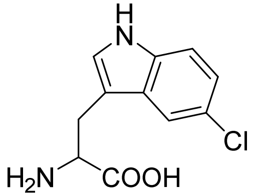
8	6-Chloro-*DL*-Tryptophan (6-ClW)	17808-21-8	abcr GmbH	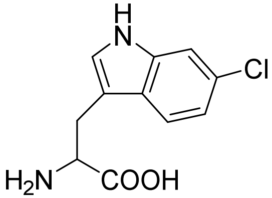
9	5-Amino-*DL*-Tryptophan (5-NH_2W)	6383-69-3	Biosynth	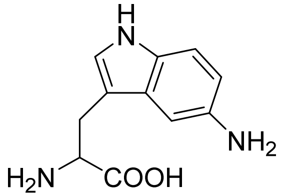
10	3-(2-Naphthyl)-*L*-Alanine (2-NaA)	14108-60-2	abcr GmbH	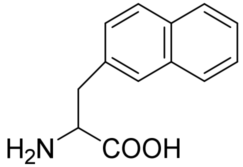
11	5-Methoxy-*DL*-Tryptophan (5-MeOW)	28052-84-8	Biosynth	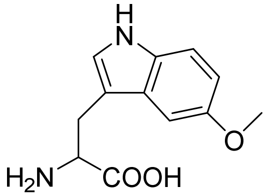

12	5-Methoxy-*L*-Tryptophan (5-MeOW)	25197-96-0	Biosynth	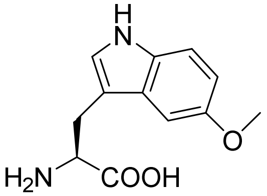
13	4-Benzoyl-*L*-Phenylalanine (Bpa)	104504-45-2	BACHEM	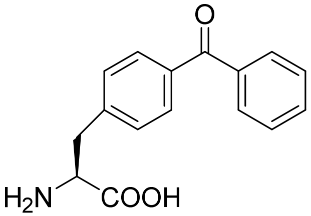
14	4-Methyl-*DL*-Tryptophan (4-MeW)	1954-45-6	Biosynth	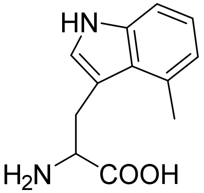
15	5-Methyl-*DL*-Tryptophan (5-MeW)	951-55-3	Merck & Sigma	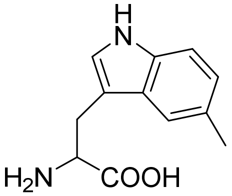
16	6-Methyl-*DL*-Tryptophan (6-MeW)	2280-85-5	Biosynth	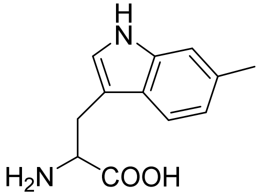

17	7-Methyl-*DL*-Tryptophan<br>(7-MeW)	17332-70-6	Biosynth	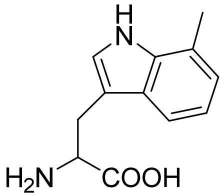
18	7-Chloro-*L*-Tryptophan<br>(7-ClW)	153-97-9	Biosynth	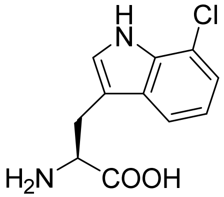
19	1-Methyl-*DL*-Tryptophan<br>(1-MeW)	21339-55-9	Merck & Sigma	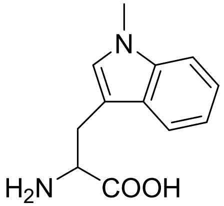
20	2,5-Difluoro-*DL*-Phenylalanine	32133-38-3	Biosynth	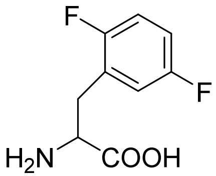
21	2,6-Difluoro-*DL*-Phenylalanine	32133-39-4	Biosynth	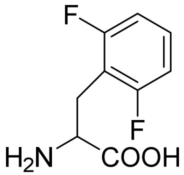
22	3,5-Difluoro-*DL*-Phenylalanine	32133-37-2	Biosynth	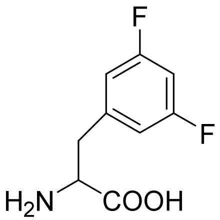

23	3,4-Difluoro-*DL*-Phenylalanine	32133-36-1	Alfa Aesar	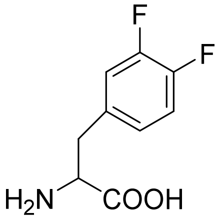
24	2,3-Difluoro-*DL*-Phenylalanine	236754-62-4	abcr GmbH	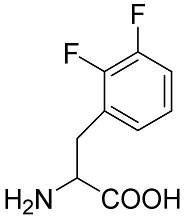
25	3-(2-Naphthyl)-*L*-Alanine (1-NaA)	55516-54-6	BACHEM	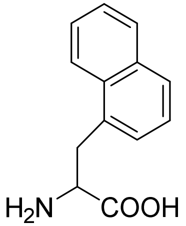
26	3,4-Difluoro-*DL*-Phenylglycine	225641-94-1	abcr GmbH	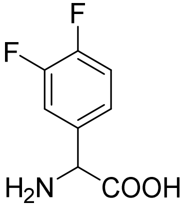
27	4-Trifluoromethyl-*L*-Phenylalanine (4-CF_3W)	114926-38-4	Alfa Aesar	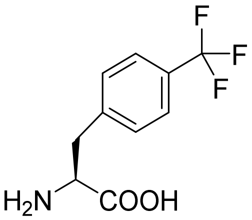

28	3-Fluoro-*DL*-Tyrosine<br>(3-FluY)	403-90-7	Alfa Aesar	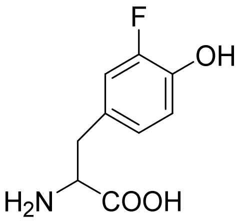
29	2,3,5,6-Tetrafluorotyrosine	157807-84-6	Merck & Sigma	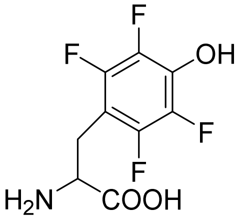
30	H-4-(7-Hydroxycoumarin-4-yl)-Abu-OH<br>(Coumarin)	905442-42-4	BACHEM	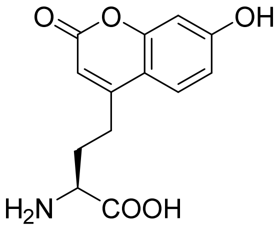
31	3-Benzothienyl-*L*-Alanine<br>(Bta)	72120-71-9	BACHEM	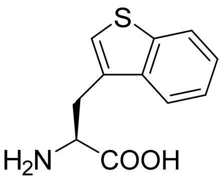

32	3-Chloro-L-Tyrosine (3-ClY)	7423-93-0	Alfa Aesar	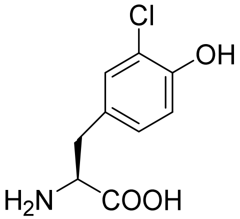
33	3-Bromo-L-Tyrosine (3-BrY)	38739-13-8	abcr GmbH	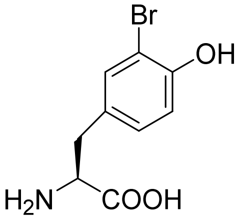
34	3,5-Dichloro-L-Tyrosine (3,5-diClY)	15106-62-4	abcr GmbH	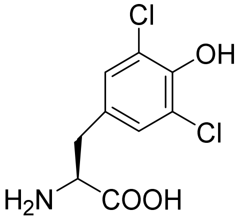
35	3,5-Dibromo-L-Tyrosine (3,5-diBrY)	300-38-9	BACHEM	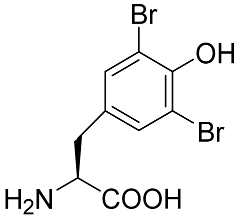

36	β-(1-Azulenyl)-L-Alanine (AzAla)	273408-71-2	It was synthesized by our lab as reported previously [6,7]	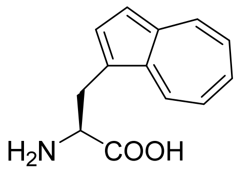
37	2-Amino-4-(Methylsulfinyl)Butanoic acid (Methionine sulfoxide)	62697-73-8	Alfa Aesar	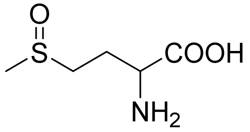
38	4-Fluoro-DL-Tryptophan (4-FluW)	25631-05-4	Biosynth	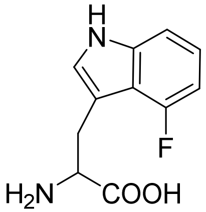
39	5-Fluoro-DL-Tryptophan (5-FluW)	154-08-5	abcr GmbH	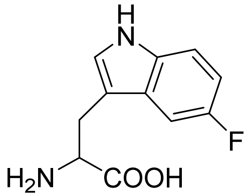

40	5-Nitro-DL-Tryptophan (5-NO_2W)	6525-46-8	Biosynth	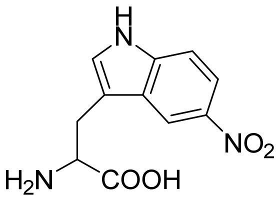
41	4-Nitro-DL-Phenylalanine (4-NO_2F)	2922-40-9	BACHEM	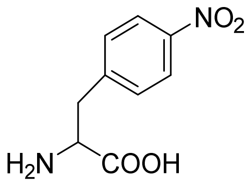
42	DL-7-Azatryptophan (7-AzaW)	7303-50-6	abcam	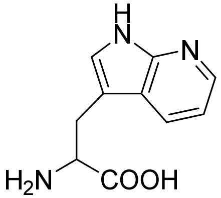
43	3,4-Dihydroxy-L-Phenylalanine (L-Dopa)	59-92-7	Merck & Sigma	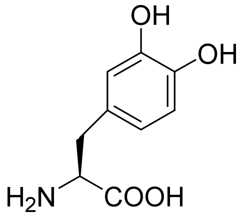

44	4-Cyano-L-Phenylalanine (4-CNF)	167479-78-9	Alfa Aesar	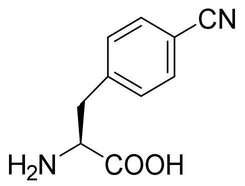
45	4-Azido-DL-Phenylalanine (4-N_3F)	33173-35-4	BACHEM	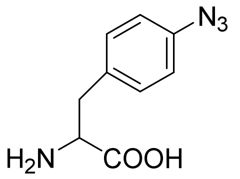
46	4-Amino-L-Phenylalanine (4-NH_3F)	943-80-6	Alfa Aesar	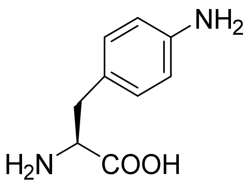
47	3-Amino-DL-Tyrosine (3-NH_2Y)	23279-22-3	BACHEM	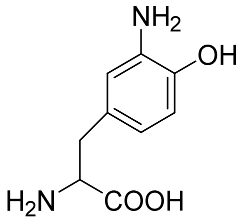

48	3-Nitro-DL-Tyrosine (3-NO_2Y)	621-44-3	Alfa Aesar	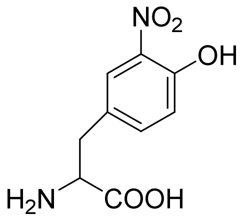
49	3-Methyl-L-Tyrosine (3-MeY)	6230-11-1	Alfa Aesar	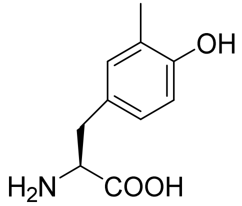
50	3-Iodo-L-Tyrosine (3-IodY)	70-78-0	TCI	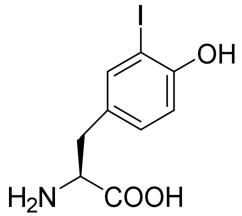

Reference

42. Muzika, M.; Muskat, N.H.; Sarid, S.; Ben-David, O.; Mehl, R.A.; Arbely, E. Chemically-defined lactose-based autoinduction medium for site-specific incorporation of non-canonical amino acids into proteins. *RSC Adv.* **2018**, *8*, 25558–25567, doi:10.1039/c8ra04359k.
63. Beyer, J.N.; Hosseinzadeh, P.; Gottfried-Lee, I.; van Fossen, E.M.; Zhu, P.; Bednar, R.M.; Karplus, P.A.; Mehl, R.A.; Cooley, R.B. Overcoming Near-Cognate Suppression in a Release Factor 1-Deficient Host with an Improved Nitro-Tyrosine tRNA Synthetase. *J. Mol. Biol.* **2020**, *432*, 4690–4704, doi:10.1016/j.jmb.2020.06.014.
65. Schumacher, D.; Lemke, O.; Helma, J.; Gerszonowicz, L.; Waller, V.; Stoschek, T.; Durkin, P.M.; Budisa, N.; Leonhardt, H.; Keller, B.G.; Hackenberger, C. P. R. Broad substrate tolerance of tubulin tyrosine ligase enables one-step site-specific enzymatic protein labeling. *Chem. Sci.* **2017**, *8*, 3471–3478, doi:10.1039/C7SC00574A.
66. Loidl, G.; Musiol, H.J.; Budisa, N.; Huber, R.; Poirot, S.; Fourmy, D.; Moroder, L. Synthesis of [beta]-(1-azulenyl)-L-alanine as a potential blue-colored fluorescent tryptophan analog and its use in peptide synthesis. *J. Pept. Sci.* **2000**, *6*, 139–144.
67 Aerni, H.R.; Shifman, M.A.; Rogulina, S.; O'Donoghue, P.; Rinehart, J. Revealing the amino acid composition of proteins within an expanded genetic code. *Nucleic Acids Res.* **2015**, *43*, 8, doi:10.1093/nar/gku1087.
68 Inokijchi, H.; Kodaira, M.; Yamao, F.; Ozeki, H. Identification of transfer RNA suppressors in Escherichia coli: II. Duplicate genes for tRNA2Gln. *J. Mol. Biol.* **1979**, *132*, 663–677, doi:10.1016/0022-2836(79)90381-4.
69 Ogawa, A.; Hayami, M.; Sando, S.; Aoyama, Y. A Concept for Selection of Codon-Suppressor tRNAs Based on Read-Through Ribosome Display in an In Vitro Compartmentalized Cell-Free Translation System. *J. Nucleic Acids* **2012**, *2012*, 7, doi:10.1155/2012/538129.
